# Genome-wide association analysis of 101 accessions dissects the genetic basis of shell thickness for genetic improvement in Persian walnut (*Juglans regia* L.)

**DOI:** 10.1186/s12870-022-03824-1

**Published:** 2022-09-13

**Authors:** Jiangtao Wang, Hang Ye, Huijuan Zhou, Pengpeng Chen, Hengzhao Liu, Ruimin Xi, Gang Wang, Na Hou, Peng Zhao

**Affiliations:** 1 Key Laboratory of Resource Biology and Biotechnology in Western China, Ministry of Education, College of Life Sciences, Northwest University, Xi’an, 710069 Shaanxi China; 2 College of Forestry, Northwest A&F University, Yangling, 712100 China; 3 Guizhou Academy of Forestry, Guiyang, 550005 Guizhou China

**Keywords:** *Juglans regia*, GWAS, Fruit-related traits, Shell thickness

## Abstract

**Background:**

Understanding the underlying genetic mechanisms that drive phenotypic variations is essential for enhancing the efficacy of crop improvement. Persian walnut (*Juglans regia* L.), which is grown extensively worldwide, is an important economic tree fruit due to its horticultural, medicinal, and material value. The quality of the walnut fruit is related to the selection of traits such as thinner shells, larger filling rates, and better taste, which is very important for breeding in China. The complex quantitative fruit-related traits are influenced by a variety of physiological and environmental factors, which can vary widely between walnut genotypes.

**Results:**

For this study, a set of 101 Persian walnut accessions were re-sequenced, which generated a total of 906.2 Gb of Illumina sequence data with an average read depth of 13.8× for each accession. We performed the genome-wide association study (GWAS) using 10.9 Mb of high-quality single-nucleotide polymorphisms (SNPs) and 10 agronomic traits to explore the underlying genetic basis of the walnut fruit. Several candidate genes are proposed to be involved in walnut characteristics, including *JrPXC1*, *JrWAKL8*, *JrGAMYB*, and *JrFRK1*. Specifically, the *JrPXC1* gene was confirmed to participate in the regulation of secondary wall cellulose thickening in the walnut shell.

**Conclusion:**

In addition to providing considerable available genetic resources for walnut trees, this study revealed the underlying genetic basis involved in important walnut agronomic traits, particularly shell thickness, as well as providing clues for the improvement of genetic breeding and domestication in other perennial economic crops.

**Supplementary Information:**

The online version contains supplementary material available at 10.1186/s12870-022-03824-1.

## Background

A comprehensive elucidation of the underlying mechanisms of crop domestication processes that drive phenotypic evolution is essential for enhancing the efficacy and feasibility of genetic level crop improvement. However, relevant studies have been more focused on annual crops, such as cereals [1], soybean [2], and tomato [3], whereas perennial crops were once regarded as intractable systems due to their larger plant size, longer juvenile phase, and generation times. Recently, the development of perennial crops has garnered increasing interest, as they are essential components for sustainable agriculture that provide alternative food sources, while lowering climate impacts [4] in the context of global warming, water shortages, rapid population growth, and environmental degradation.

There are four sections (sect. Dioscaryon, Cardiocaryon, Trachycaryon, and Rhysocaryon) in the genus *Juglans*, comprising approximately 20 perennial species extensively distributed across the planet [5]. Owning to its edible and highly nutritious fruit, English or Persian walnut (*Juglans regia* L.) is one of the oldest food sources known, as well as the most commercially significant *Juglans* species cultivated for nut production [5]. It is a monoecious tree species that contains 2n = 2x = 32 chromosomes, which are primarily grown in the temperate regions of Europe, North and South America, South Africa, Asia, Australia, and New Zealand [6]. The historical cultivation and domestication of the Persian walnut can be traced back 6800 years, with the cradle of its domestication thought to be in Central Asia [7]. According to the Food and Agriculture Organization of the United Nations, the global in-shell walnut production (mostly from China, the United States, and Iran) was more than 14,800 t (https://www.fao.org/faostat/zh/#search/walnut). China is presently viewed as one of the primary walnut diversity centers, providing abundant germplasm sources for walnut cultivating and breeding. Thus, as a leading walnut producer, the quality of walnut fruit is related to the selection of traits such as thinner shells, larger filling rates, and improved taste, which is very important for its breeding and procreation in China [6, 8, 9].

Investigations into the improvement of genetic walnut breeding have a long developmental timeline. As a preliminary breeding approach, morphological research has provided directions for the selection of cultivars that are suited to specific growing conditions [10]. Correlations between various horticultural or agronomic traits were elucidated in the early stages [7]. Furthermore, the emergence of advanced molecular investigative tools has enabled new insights into selection processes and evolutionary trajectories. The development of high-density DNA markers has played a significant role toward understanding the genetic diversity of different germplasms, while accelerating breeding programs and selection efficiencies for complex quantitative traits in plants. Various classes of molecular markers including AFLPs [11], SSRs [12], and SNPs [13] have been adopted by researchers involved in genetic mapping, genotype characterization, genetic diversity, and relationship determination in walnut.

To date, thanks to the improvement of next-generation sequencing (NGS) technologies, it is possible to detect the genome-wide variations in a considerably short time, which makes single-nucleotide polymorphism (SNP) markers an ideal choice. The release of the first reference genome of *J. regia* through de novo assembly from short reads opened and subsequently extended the genomic era for studies on walnut evolution and enhancements in genetic breeding. With the assistance of up-to-date long-read and Hi-C sequencing technologies, higher-quality reference genomes of the *J. regia* have since been published [14]. These have laid a groundwork for investigating the genetic mechanisms that underlie the optimal walnut agronomic and crop traits such as expanded yields, larger nut sizes, thinner shells, and light kernel color.

Against this background, genome-wide association studies (GWAS) as an ideal alternative tool can capitalize on linkage disequilibrium (LD) in diverse populations to single out target quantitative trait loci. Compared with traditional linkage mapping methods, GWAS has several advantages, as it takes on natural variation and has a higher genetic resolution by exploiting historical recombination [15]. The *J. regia* 700 K SNP array developed from the resequencing data (27 founders) of the Walnut Improvement Program (WIP) of California University, USA, has enormously promoted the research on the genetic level and development of walnut breeding [16]. Using this SNP panel as a backdrop, researchers have performed GWASs to unearth genomic loci linked to fruit phenotypes and phenology-related traits [6, 8, 9], nut-related traits [17], and water-use efficiencies [18]. A few associated SNPs in view of these discoveries have been applied further to develop molecular markers to select accessions with desirable target traits [9, 19].

Nevertheless, due to the technical limitations of the SNP array, although the associated loci can be identified, it is typically difficult to determine the causative genes and variations responsible for given traits. It is still unknown how genomes and genes change during the cultivation of the long-lived perennial walnut crop. Conversely, in addition to the few existing studies that explored limited association gene loci focused on specific given traits, the genetic basis of various important agronomic traits remains for researchers to decipher. For example, in addition to the edible fruit and the usual fruit-related traits, the walnut shell, which is discarded after the walnut is consumed, is an excellent industrial raw material [20]. Walnut shells are mainly composed of lignin and cellulose, which can be used in stone grinding, the asphalt felt industry, and activated carbon production [21]. Moreover, it is well known that cellulose, which is used to meet the high daily demand for paper in China, is a good material for manufacturing paper [22].

Thus, in this study, for the first time, we investigated the genetic control of the cellulose content of walnut shells. Firstly, a total of 101 Persian walnut cultivars, distributed across Guizhou Province (an important walnut growing center in China), were collected for whole-genome resequencing. Meanwhile, the shell thickness, combined with nine other fruit-related traits (for a total of 10 traits), were measured for the follow-up GWAS. Lastly, the expression levels of candidate genes corresponding to different tissues and development stages in walnut were further verified through transcriptomic analysis and qRT-PCR experiments. The aims of this study were (1) to understand the genetic structures and relationships of the walnut populations under study and their linkage disequilibria, and (2) to identify correlations between molecular markers and traits using GWAS to aid with marker-assisted breeding. In addition to providing considerable available genetic resources for walnut trees, this study revealed the underlying genetic foundations involved in important agronomic traits, particularly walnut shell thickness, in addition to offering insights into the improvement of genetic breeding and domestication for other perennial commercial crops.

## Methods

### Sample collection and measurement of agronomic traits

This study was approved by the Chinese government and carried out according to the laws of the People’s Republic of China. All participants had a license approval letter from the College of Life Sciences, Northwest University. From 2020 to 2022, when the green husk cracked in September, 101 Persian walnut accessions in 12 genotypes were sampled in Guizhou Province, China, and investigated. The accessions sampled in the same location were regarded as belonging to one genotype category. The number of trees for each genotype ranged from five to twelve (Table S1). Each individual was collected at least 30 m from the others to prevent the selection of clones. Additionally, for each accession, five fruits (biologically replicates) were adopted in further trait measurements. The collected samples were identified by the author Hou Na (professor of botany at Guizhou Academy of Forestry) according to their phenotypes. All samples were stored at the Walnut Research Institute, Guizhou Academy of Forestry, Guiyang, Guizhou (106°44′ E, 26°29′ N) with the voucher specimen accession numbers JR0001–JR0101 (Table S1). Young fresh leaves were rinsed clean with distilled water, frozen in liquid nitrogen, and stored at − 80 °C for pending DNA extraction.

Subsequently, eight quantitative traits of interest that described the walnut fruits were measured, namely, longitudinal diameter (LD), cross diameter (CD), side diameter (SD), single-nut weight (SW), kernel weight (KW), shell thickness (ST), fat content (FC), and protein content (PC). Specifically, the LD, CD, and SD were measured using an electronic Vernier calliper (accuracy 10^− 2^ mm), while an electronic balance (METTLER TOLEDO ME104) was employed to evaluate the SW and KW (accuracy of 10^− 1^ g). Each accession within five biologically replicates was used to measure these traits, and the standard deviation (SD) of these repetitions was calculated (Table S2). Additionally, Coomassie Brilliant Blue G-250 (Solarbio-C8420) [23] was employed to estimate the PC. Coomassie blue staining is a kind of dye-binding method. In the free state, the stain is red with the maximum light absorption of 488 nm. When it becomes cyan after binding to the protein, the protein–pigment conjugate has maximum light absorption at a wavelength of 595 nm. Its light absorption value is proportional to the protein content; therefore, it can be used for the quantitative determination of proteins. FC was assessed following Soxhlet extraction methods (Table S2) [24], which is a method of extracting compounds from solid substances, widely used in the determination of fats. Furthermore, two index traits (fruit index (FI) and filling rate (FR)) were calculated using the following formulas: FI = CD/LD and FR = KW/SW (GBT20398–2021), respectively. Lastly, a total of 10 traits were further applied in the GWAS. The coefficients of variation and correlation of the phenotypes were calculated using the R package “GGally” and visualized using “ggplot2” [25]. The R package FactoMineR (http://factominer.free.fr/) was used to conduct the PCA (principal component analysis). Additionally, the NJ (neighbor-joining algorithm) and UPGMA (unweighted pair-group method with arithmetic means) methods were employed to perform the cluster analysis as a function of the Euclidean distances of these studied traits.

### DNA extraction and whole-genome resequencing

Genomic DNA was extracted from fresh walnut leaves using a modified CTAB technique [26], followed by the determination of its quality and concentration using an ultraviolet spectrophotometer (MAPADA LIV-3200) and agarose gel electrophoresis (0.8%), respectively. The insert size libraries (~ 350 bp) were constructed from randomly fragmented genomic DNA, which was dissociated using a Covaris ultrasonic shearer. A Truseq Library Prep Kit (Illumina) was employed to build the library using the following steps according to Illumina’s standard protocol: end repair, polyA tail addition, sequencing connector addition, purification, and PCR amplification. Next, 150 bp paired-end reads were produced using the Illumina HiSeq 2500 sequencing platform (Illumina, San Diego, CA,).

### SNP calling and genotyping

FASTP software was adopted to filter the raw data using default parameters. Subsequent to pruning, the clean reads were aligned to the chromosome-level reference genome (*J. regia* Genome V1.0) using the Burrows–Wheeler alignment maximal exact match (BWA-MEM) algorithm, with parameters -T 20 -k 30 [27]. The alignment results, i.e., the SAM files that corresponded to each sample, were transferred and sorted using SAMTOOLS [28], which was also employed to calculate the alignment rates and depths, respectively. We then used PICARD [29] to remove duplicates followed by local realignment utilizing GATK [30]. Subsequently, SNP calling was conducted using the joint calling strategy in GATK. Firstly, we obtained the genomic variant call format (GVCF) for each sample, and then carried out joint variant calling using the module “CombineGVCFs”. The combined GVCF file was finally used to detect the variants via the “GenotypeGVCFs” module. The vcfutils.pl script package in BCFTOOLS [31] was preliminarily employed to filter SNPs with the parameters -w 5 -W 10, in which SNPs within 5 bp around a gap were filtered with a window size of 5 bp. Furthermore, the “VariantFiltration” module in GATK was implemented to filter the SNPs using the following the filtering expression: QUAL< 30 || QD < 2.0 || FS > 60.0 || MQ < 40.0, where QUAL, QD, FS, and MQ represented the quality, quality by depth, *p*-value of Fisher’s exact test, and mapping quality, respectively. PLINK [32] software was used to perform subsequent pruning by removing SNPs with minor allele frequencies (MAF) ≤0.5 and missing genotype rates < 20%. Consequently, a total of 10,930,190 (Table S3) high-quality SNPs remained for further analysis after filtering from 24,715,804 raw SNPs.

### Population structure and LD analysis

To explore the phylogenetic relationships of the studied walnut accessions, we constructed a neighbor-joining (NJ) tree using MEGA software [33], in which the Kimura two-parameter mode was adopted, with the site coverage cutoff value set to 80%. Principal component analysis (PCA) was also implemented to elucidate the genetic structure using GCTA software [34]. Additionally, a more precise population genetic structure was inferred using ADMIXTURE software [35]. Notably, one of the major assumptions employed in inferring population structures was that there were no spurious correlations between the measured variables. Consequently, the physical and linkage disequilibrium-correlated SNPs needed to be pruned prior to population structure estimation. The SNPs contained an *r*^*2*^ value > 0.2 within the 50 kb window size, and 10 bp step sizes were removed, leaving 10,689,600 SNPs for ADMIXTURE analysis. The prior genetic cluster value (*K*) was set from 1 to 5. The optimal *K* value was inferred according to the maximum marginal likelihood value according to minimum cross-validation (CV) errors.

Linkage disequilibrium (LD) is the nonrandom association of alleles at different loci. To evaluate the LD patterns among the studied accessions, LD decay was computed using POPLDDECAY software [36]. Nonrandom associations between alleles at different loci were measured by *r*^2^. The visualization of the results was performed using the Plot_MultiPop.pl script package in POPLDDECAY.

### GWAS mapping and haplotype networking

Before carrying out the GWAS, the outlier values of the phenotypic data were removed. GWAS was performed using GEMMA [2], which accounted for the familial relationships in the form of a kinship matrix obtained using the centered IBS method. Following the model comparison, the mixed linear model (MLM), where Q (population structure) and K (kinship matrix) were fitted to the model as fixed and random effects, respectively, to reduce type I errors due to spurious associations from relatedness and population structure, was finally adopted in this study. For details, the MLM model used the following formula: *y* = *Xα* + *Zβ* + *Wμ* + *e*, where *y* is the phenotypic trait, *X* is the indicator matrix of fixed effects, *Z* is the indicator matrix of SNPs, *W* is a matrix for random effects, and *e* is the random residual subjected to e ≈ (0, δ2). Candidate SNPs were initially determined by the 5% significance threshold (the −log_10_ of the *p-*value was > 5) through the correlation results. In addition, the Bonferroni correction method was employed to correct the *p-*value via the multiple hypothesis test, to reduce the probability of false positives. Candidate SNPs were initially determined by the 5% significance threshold (the −log_10_ *p* ≥ 5) through the correlation results. The Q–Q and Manhattan plots were constructed to better interpret the association results using the R package “CMplot” (https://github.com/YinLiLin/R-CMplot).

### Candidate gene identification and enrichment analysis

To locate the candidate genes, LD blocks around the most correlated loci were estimated using LDBLOCKSHOW software [37]. Each physical position of these trait–SNP associations was investigated to explore the extension of the surrounding LD blocks, as well as to identify the genomic regions to search for candidate genes. The LD blocks were investigated using the method of Gabriel et al. with default parameters [38]. The identified LD blocks were then surveyed for candidate genes via mapping to the reference genome. Subsequently, these identified candidate genes were annotated using EGGNOG-MAPPER [39]. To better understand the functions of the candidate genes, TBTOOLS [40] was adopted to execute GO and KEGG enrichment analysis, in which the annotation of the whole reference genome was regarded as the background. Furthermore, the Bonferroni correction method [41] was adopted to detect the significant GO terms or KEGG pathways.

### Transcriptome profile analysis and qRT-PCR verification of the candidate *JrPXC1* gene

According to the GWAS results, *JrPXC1* was identified as a significant candidate gene that mediated the thickness of walnut shells, which encoded a leucine-rich repeat protein kinase PXC1 (see results). A total of 17 walnut transcriptome datasets obtained from previous studies [42] were employed to explore the expression levels of the *JrPXC1* gene in different tissues including immature fruit, pistillate flower, embryo, somatic embryo, vegetative bud, callus exterior, catkins, hull cortex, hull immature, hull peel, young leaves, and roots (Table S4). Moreover, using other published transcriptome datasets [43], the expression of the *JrPXC1* gene in the green leaves and husks of walnut at three development stages (each stage included three replications) was investigated (Table S4). In the transcriptome analysis, the raw data were filtered by FASTP [44], and the clean reads were aligned to the reference genome using HISAT2 [45]. The gene expression was then calculated using FEATURECOUNTS [46].

To verify the expression pattern of *JrPXC1* in the walnut shell and husk, we collected sample tissues (three biological replicates) from *J. regia* trees growing in Xi’an, Shaanxi Province (at Northwest University campus) from May to July at three development stages (13 May, 31 May, and 7 July) (Table S5). Using the Plant RNA Kit from OMEGA (USA), the total RNA was isolated from frozen leaf powders. A NanoDrop 2000 spectrophotometer was used to verify the quality and quantity of the RNA samples. According to the manufacturer’s instructions, a sample of the total RNA (1 μg) was reverse-transcribed for first-strand cDNA synthesis using a PrimeScript™ RT Master Mix (Takara). Gene-specific primers were designed using Primer3Plus (https://www.primer3plus.com/index.html) according to the sequence of the target gene in the reference genome. After 1:5 dilution using RNase-free water, synthetic cDNA was employed as the qRT-PCR template. The qRT-PCR was performed using a Bio-Rad CFX96 Touch Real-Time PCR system and BioTeKe 2 × Plus SYBR real-time PCR mixture. For the internal reference gene, walnut *β*-actin was used as an internal reference gene.

## Results

### Phenotypic diversity and genetic variations

**Fig. 1:**
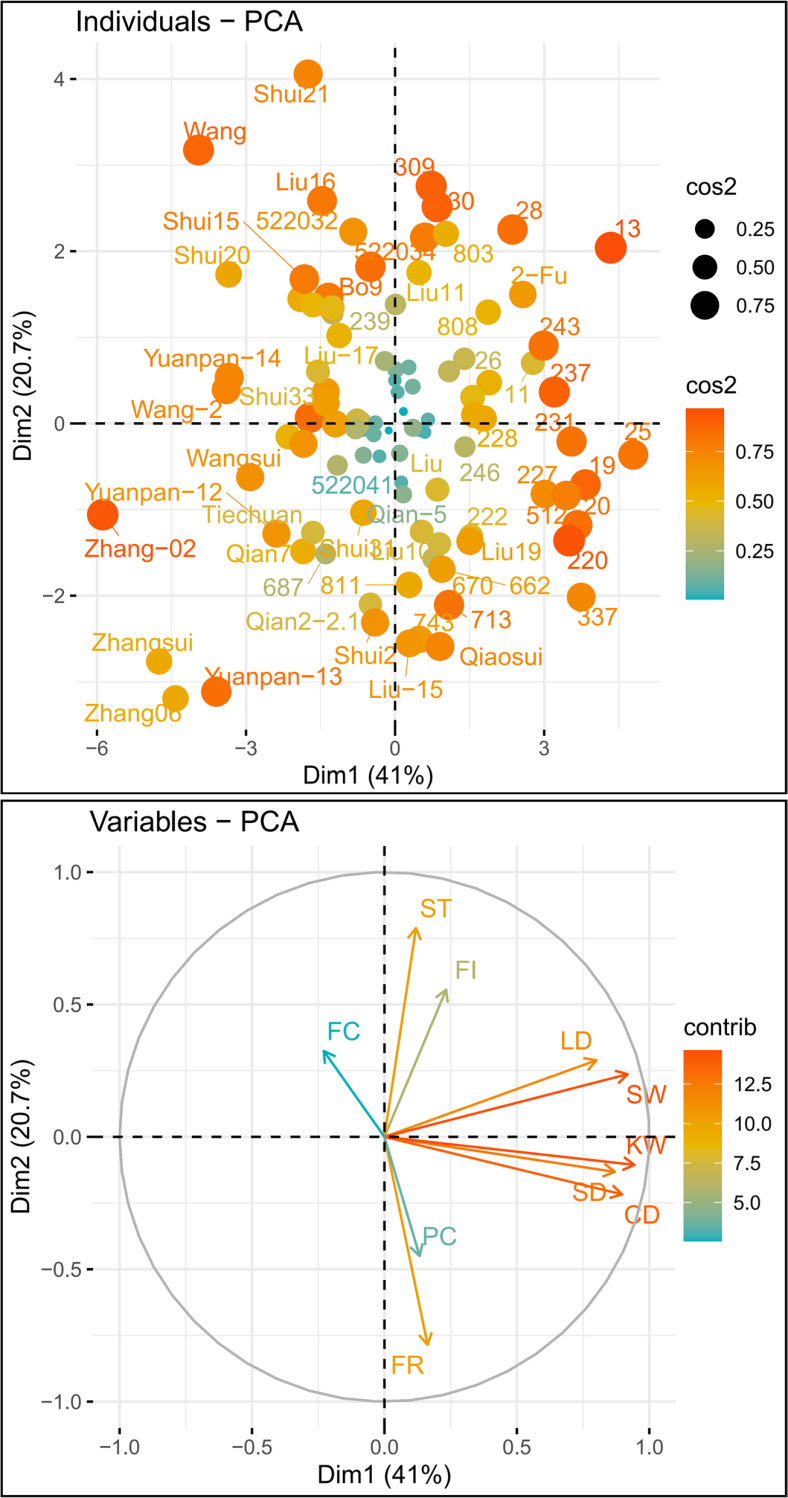
Principal component analysis (PCA) biplot of ten phenotypic traits within 101 walnut individuals

For the phenotypes, the frequency distribution histogram of phenotypic measurement results indicated that seven traits (CD, SD, SW, FR, ST, FC, and PC) of the 101 walnut individuals presented a normal distribution, while the other three phenotypic data (LD, FI, and KW) showed a skewed normal distribution pattern (Figs. 1 and S1). The mean values of LD, CD, and SD were 34.07 mm, 29.69 mm, and 32.07 mm, respectively (Table S2). The values of ST, SW, and KW ranged from 0.70 mm to 2.16 mm, from 3.19 g to 15.63 g, and from 1.69 g to 8.86 g, with average values corresponding to 1.28 mm, 9.30 g, and 4.75 g, respectively. The distribution patterns of ST values were mainly concentrated; however, they fluctuated significantly for SW and KW. Additionally, the maximum SW value was 16 g, while the minimum KW value was 1.6 g. The mean percentage of FC (67.56%) was 3.54 times higher than that of PC (19.05%). Interestingly, the percentage of FC was significantly negatively related to PC according to the correlation analysis results (Fig. 1; Table S2). It was observed that the three phenotypes related to fruit size (LD, SD, and CD) were positively correlated to SW and KW. Furthermore, a negative correlation was found between FR and CD, whereas ST and SW were positively correlated. CD also exhibited a significant negative correlation with FR (Fig. 1; Table S2).

For the genotypes, a total of ~ 906.1 Gb clean reads were generated from whole-genome resequencing with an average read depth of 13.80× for each accession. The mean Q30 (sequencing error rate < 0.05%) ranged from 90.92 to 93.18% across all studied individuals, with an overall mean Q30 of 92.03%. The GC ranged from 37.72 to 40.61%, whereas the mean GC was 38.87% (Table S1). The average mapping rate was 93.69% with a mean coverage (4×) of 86.14% (Table S1). The SNP numbers for each individual were counted and compared with the reference genome, and the results showed that the individual “662” contained the most SNPs (23,521,885), while the individual “TieChuan” presented a relatively low variability, containing the lowest number of SNPs (12,225,075) (Table S1). A total of 45,548 SNPs remained for further analysis after filtering from 4,343,085 raw SNPs. The results of the SNP annotation revealed that most SNPs resided in the intergenic regions (7,632,645), and the nonsynonymous/synonymous substitution ratio for the SNPs in the exonic regions was 1.50 (Table S3). The densities of SNPs and InDels along the chromosome are shown with a 1 Mb window size (Figs. S2 and S3).

### Population structure and linkage disequilibrium (LD)

**Fig. 2:**
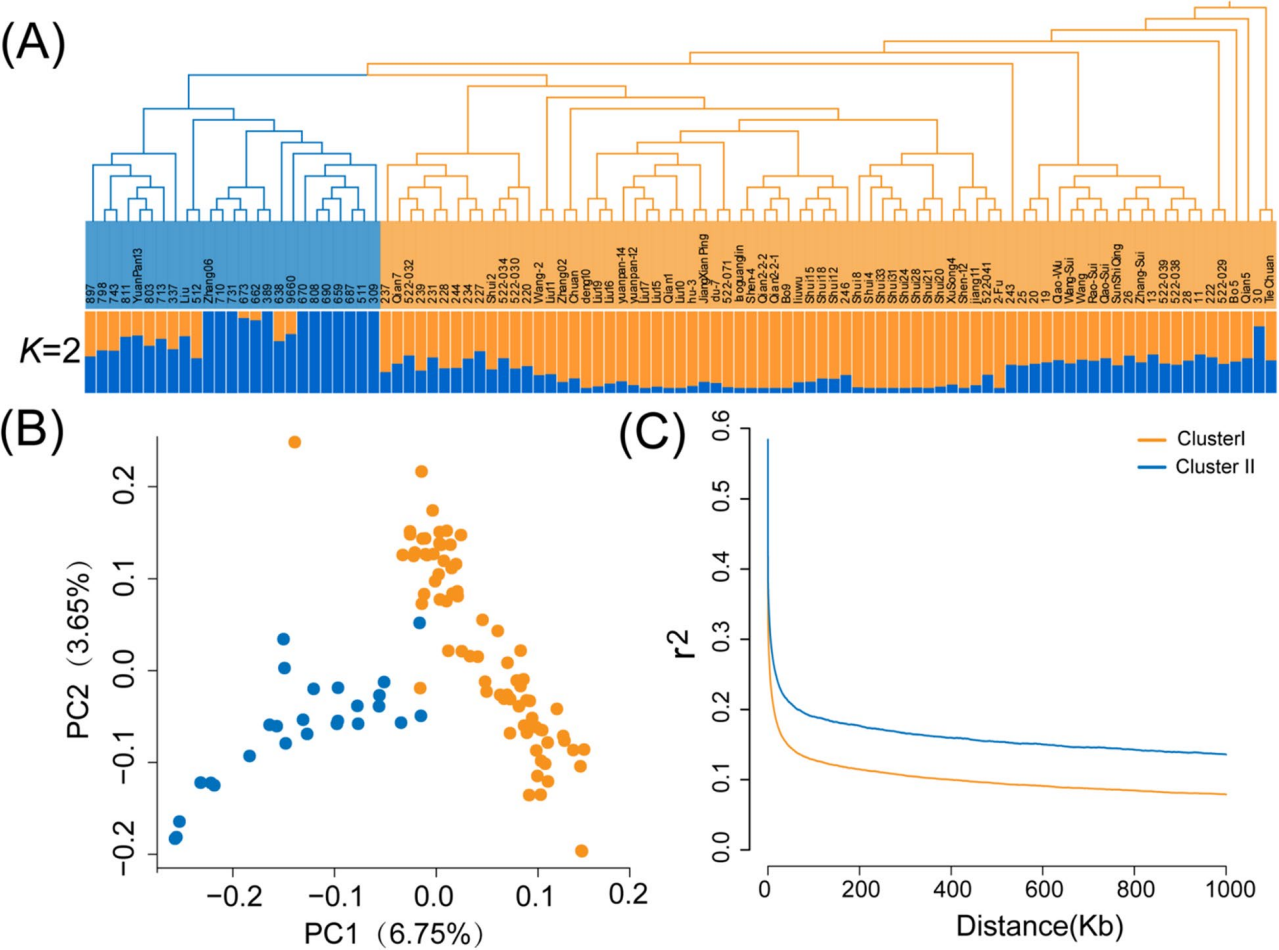
Population structure and linkage disequilibrium (LD). **A** Neighbor-joining tree and population structure of 101 accessions; **B** Principal component analysis (PCA); **C** Linkage disequilibrium (LD) analysis

Clustering analysis distinguished all individuals into two major clusters using the NJ tree with 1000 bootstrap replicates. Cluster I contained 25 individuals (blue), while 76 individuals (orange) were classified into Cluster II, which corresponded with the results of ADMIXTURE analysis (Fig. 2). In ADMIXTURE, the lowest CV error of 0.536 indicated that the optimal *K* value was 2, which revealed that the most significant possibilities of population structure were classified into two groups. The genetic structures corresponding to *K* values of 3 and 4 were also determined, with the results showing a similar pattern with *K* = 2, i.e., more individuals were mixed within the established populations (Fig. S4). Additionally, the results observed from PCA analysis were engaged with NJ tree or ADMIXTURE. The first two principal coordinates detected 10.4% of the total variation, in which PC1 and PC2 accounted for 6.75 and 3.65% of the genetic variation, respectively (Figs. 2 and S5). However, the results of phenotypic cluster analysis showed an inconsistent pattern when compared to the genetic cluster analysis (Fig. S6). The phenotypic clusters were not significantly correlated to the genetic cluster. The individuals belonging to genetic Cluster I and Cluster II mixed together and did not form distinct clades.

Furthermore, LD analysis was performed to access the intensity of linkages between the studied accessions. LD was significantly variable in different populations, while it was divided by structural analysis (Fig. 2). The average attenuation distances at which LD values decayed below half the maximum value of *r*^*2*^ were 3.8 kb and 7.1 kb for Cluster I and Cluster II, respectively. The rate of LD decay for Cluster I was much quicker than that for Cluster II, indicating that the LD in Cluster II was stronger than that in Cluster I.

### Genome-wide association study (GWAS)

The GWAS was based on the MLM model, where a total of 12,105 SNPs over the threshold (−log_10_ *p* ≥ 5; 5% Bonferroni threshold) were initially considered as candidate loci accounting for the studied phenotypic traits (Fig. S7; Table S6). Among the studied traits, KW contained the most number (1979) of candidate SNPs, while FC was associated with the lowest number of candidate SNPs (379). Chromosome 11 contained the greatest number of candidate SNPs (2021), followed by chromosome 4 (1866) and chromosome 3 (1325). Chromosome 12 contained the lowest number of candidate SNPs at only 227. Furthermore, combined with LD block analysis, 1212 SNPs located in 438 identified LD blocks were adopted to explore the candidate genes that accounted for these phenotypic traits. The number of the LD blocks ranged from 13 to 73, which corresponded to the FC and KW traits, respectively. Chromosome 11 contained the largest number of candidate blocks (52), while there were only 15 candidate blocks distributed on chromosome 12. Chromosomes 3 and 11 contained blocks for all studied traits, where chromosome 11, in particular, was the most significant chromosome associated with the studied traits including LD (eight LD blocks for 154 candidate SNPs), SD (nine LD blocks for 35 SNPs), SW (eight LD blocks for 23 SNPs), and KW (seven LD blocks for 172 SNPs).

These identified LD blocks were used to further explore the candidate genes that possessed the specific functions that responded to the variations of the studied traits. There were a total of 329 candidate genes identified as signals that accounted for the phenotypic variations. GO and KEGG enrichment analyses were conducted for these candidate genes, with the results showing that they were significantly enriched in three GO terms that corresponded to molecular functions (95 genes), cellular components (113 genes), and biological processes (112 genes) (Fig. S8). For the molecular functions, six genes were involved in “glucosyltransferase activity”, and seven genes were related to “hexosyltransferase activity”. For the cellular components, 10 specific GO terms, namely, “plant-type vacuole”, “extracellular space”, “obsolete extracellular region part”, “vacuole”, “plant-type vacuole membrane”, “plant-type cell wall”, “extracellular space”, “cell wall”, “external encapsulating structure”, “cell periphery”, and “membrane” were enriched among 113 candidate genes. There were 8, 6, 6, 18, 5, 7, 9, 10, 10, 40, and 54 candidate genes significantly enriched in the biological processes of “pollen development”, “gametophyte development”, “external encapsulating structure organization”, “organic hydroxy compound biosynthetic process”, “organic hydroxy compound metabolic process”, and “monocarboxylic acid metabolic process”, respectively. Moreover, the results of KEGG enrichment analysis indicated that these candidate genes were significantly enriched in five KEGG pathways, namely, “BRITE hierarchies” (92), “protein families: genetic information processing” (53), “metabolism” (34), “protein families: metabolism” (31), and “signaling and cellular processes” (23).

Combined with the results of gene annotation, for FR, the JreChr03G11738 and JreChr11G10011 genes, located on chromosomes 3 and 11, were considered as the relevant candidate genes (Table 1). The former was annotated as the homologous SAG39 gene, while the latter was annotated as MTP10. For the CD traits, six candidate genes resided on chromosomes 3, 4, 5, 11, and 16, which corresponded to MSP, MYB35, TOGT1, KAS3A, and RLK7, respectively. Six candidate genes, namely, IAA16, FER, ERG3, AGL19, KAS3A, and RID3, located on chromosomes 7, 11, and 16, were observed for the LD trait. Variations in PC and FI could be explained by eight genes, namely, ABC1K8, ZAR1, ABCB21, PAE12, ABC1, MED19A, MYB1R1, and ATPK2 for the former, and PCO4, KOM, ABC1, AIL5, HHT1, LRK10L-2.8, FER, and HHP2 for the latter. Thirteen (DLO1, MYB1, HAT, WAKL8, WOX3, SNE, CKP11, GGAT2, IAA16, 4CL2, CSLG3, MYB106 and DIS1), six (HAT, MTERF15, FER, ERG3, PHOS34, and FRO2) and seven (WAKL8, CKP11, NBR1, CSLG3, ERG3, TPX2, and KAS3A) candidate genes were found, which were significantly associated with the KW, SD, and SW traits, respectively. Nevertheless, there was only one candidate gene attributed to the FC trait, located on chromosome 2.

**Table 1:** Annotations and statistical data of single nucleotide polymorphisms (SNPs) significantly associated with walnut shell thickness and fruit shape traits. Note: *Chr* Chromosome, *BP* Physical Position

Phenotype	Chr	bp	Ref	Alt	-Log10 (*P*-value)	Gene ID	Description	Annotation
LD (Longitudinal diameter)								
chr7		12,074,087	A	G	6.69399875	JreChr07G10135	auxin-responsive protein IAA16	IAA16
chr11		10,149,576	T	C	7.244879262	JreChr11G10006	receptor-like protein kinase FERONIA	FER
chr11		30,756,326	A	T	6.137889799	JreChr11G11391	elicitor-responsive protein 3	ERG3
chr11		31,328,022	C	T	6.524569411	JreChr11G11443	agamous-like MADS-box protein AGL19 isoform X1	AGL19
chr11		32,159,970	C	A	5.555962234	JreChr11G11543	3-oxoacyl-[acyl-carrier-protein] synthase 3 A, chloroplastic	KAS3A
chr16		3,210,546	G	A	5.390427359	JreChr16G10833	protein ROOT INITIATION DEFECTIVE 3	RID3
CD (Cross diameter)								
chr3		9,630,490	A	G	5.583537074	JreChr03G13485	leucine-rich repeat receptor protein kinase MSP1	MSP1
chr4		1,719,010	C	T	5.350563201	JreChr04G10551	transcription factor MYB35	MYB35
chr5		920,085	G	T	5.319522344	JreChr05G12961	scopoletin glucosyltransferase	TOGT1
chr5		32,730,442	G	T	5.285313561	JreChr05G11651	Brassinosteroid insensitive 1-associated receptor kinase 1	BAK1
chr11		32,159,970	C	A	5.51601553	JreChr11G11543	3-oxoacyl-[acyl-carrier-protein] synthase 3 A, chloroplastic	KAS3A
chr16		18,020,713	T	C	5.485163743	JreChr16G10512	receptor-like protein kinase 7	RLK7
SD (Side diameter)								
chr3		40,515,063	C	T	5.505483366	JreChr03G12405	zinc finger BED domain-containing protein DAYSLEEPER	HAT
chr11		9,050,153	G	A	5.404420675	JreChr11G12632	transcription termination factor MTERF15, mitochondrial	MTERF15
chr11		10,149,576	T	C	5.028683183	JreChr11G10006	receptor-like protein kinase FERONIA	FER
chr11		30,756,326	A	T	5.552353662	JreChr11G11391	elicitor-responsive protein 3	ERG3
chr12		23,968,954	C	T	5.043208631	JreChr12G11314	universal stress protein PHOS34	PHOS34
chr13		14,692,404	G	C	5.401969681	JreChr13G10328	ferric reduction oxidase 2	FRO2
ST (Shell thickness)								
chr1		43,852,395	T	G	5.254460231	JreChr01G12569	FLG22-induced receptor-like kinase 1	FRK1
chr2		23,755,810	C	T	5.169570452	JreChr02G11142	ELF3-Like	ELF3-Like
chr2		24,412,382	C	T	5.748116357	JreChr02G11216	LRR receptor-like serine/threonine-protein kinase IOS1	IOS1
chr3		3,094,759	T	C	6.114072587	JreChr03G11591	serine/threonine-protein kinase STY13	STY13
chr3		29,892,251	G	A	5.127508605	JreChr03G11531	multiprotein-bridging factor 1b	MBF1
chr5		20,620,130	C	G	5.484158862	JreChr05G10768	snRNA-activating protein complex subunit	SRD2
chr5		26,729,080	T	C	5.657466193	JreChr05G11239	receptor protein kinase TMK1	TMK1
chr6		15,911,060	G	A	6.611432591	JreChr06G10266	cytochrome P450 86B1	CYP86B1
chr9		2,068,541	A	G	5.600622946	JreChr09G10845	protein MKS1	MSK1
chr10		349,801	C	T	6.844556769	JreChr10G11611	leucine-rich repeat receptor-like protein kinase PXC1	PXC1
chr11		13,887,811	A	G	5.07742639	JreChr11G10209	9-cis-epoxycarotenoid dioxygenase NCED1, chloroplastic	NCED1
chr11		21,239,109	C	T	5.846222929	JreChr11G10535	abscisic acid 8′-hydroxylase CYP707A2	CYP707A2
chr13		23,631,873	A	T	5.229763724	JreChr13G10891	transcription factor GAMYB	GAMYB
chr14		685,568	A	G	5.095229617	JreChr14G11517	protein IQ-DOMAIN 14	IQD14
SW (Single weight)								
chr4		7,285,962	T	A	5.305655983	JreChr04G12337	wall-associated receptor kinase-like 8	WAKL8
chr5		19,151,078	C	T	5.70994	JreChr05G10699	calcium-dependent protein kinase 11	CKP11
chr11		2,249,234	T	C	5.18404523	JreChr11G10598	protein NBR1 homolog	NBR1
chr11		28,523,493	A	C	5.173799076	JreChr11G11160	cellulose synthase-like protein G3	CSLG3
chr11		30,756,326	A	T	5.466738391	JreChr11G11391	elicitor-responsive protein 3	ERG3
chr11		31,479,411	G	A	5.417245272	JreChr11G11465	protein TPX2-like isoform X1	TPX2
chr11		32,159,970	C	A	6.042857182	JreChr11G11543	3-oxoacyl-[acyl-carrier-protein] synthase 3 A, chloroplastic	KAS3A
KW (Kernel weight)								
chr1		13,182,401	T	C	5.162116115	JreChr01G10234	protein DMR6-LIKE OXYGENASE 1	DLO1
chr3		5,831,898	G	A	6.010662241	JreChr03G13034	transcription factor MYB1	MYB1
chr3		40,515,066	C	T	5.500387414	JreChr03G12405	zinc finger BED domain-containing protein DAYSLEEPER	HAT
chr4		7,285,501	T	C	5.616759827	JreChr04G12337	wall-associated receptor kinase-like 8	WAKL8
chr4		29,960,180	G	A	5.883457671	JreChr04G11264	WUSCHEL-related homeobox 3	WOX3
chr4		37,250,235	G	A	5.858566533	JreChr04G11895	F-box protein SNE	SNE
chr5		19,151,078	C	T	5.362740588	JreChr05G10699	calcium-dependent protein kinase 11	CKP11
chr6		17,840,466	A	G	6.212932268	JreChr06G10365	glutamate--glyoxylate aminotransferase 2	GGAT2
chr7		12,074,087	A	G	5.314401916	JreChr07G10135	auxin-responsive protein IAA16	IAA16
chr7		32,680,175	G	T	5.592438471	JreChr07G11894	4-coumarate--CoA ligase 2	4CL2
chr11		28,523,493	A	C	5.100453069	JreChr11G11160	cellulose synthase-like protein G3	CSLG3
chr12		7,066,577	G	A	5.008780394	JreChr12G11745	transcription factor MYB106	MYB106
chr13		1,067,741	A	G	5.038630597	JreChr13G10052	E3 ubiquitin-protein ligase DIS1	DIS1
FC (Fat content)								
chr2		27,530,158	C	T	5.509575712	JreChr02G11641	Transcription factor bHLH103	BHLH103
PC (Protein content)								
chr3		13,319,681	T	G	5.393661529	JreChr03G10302	protein ACTIVITY OF BC1 COMPLEX KINASE 8, chloroplastic	ABC1K8
chr5		9,505,823	A	G	5.251919009	JreChr05G13008	receptor protein kinase-like protein ZAR1	ZAR1
chr7		26,479,884	G	A	6.244650196	JreChr07G11142	ABC transporter B family member 21	ABCB21
chr7		26,655,396	C	T	5.047106959	JreChr07G11161	pectin acetylesterase 12-like	PAE12
chr7		26,655,396	C	T	5.047106959	JreChr07G11162	protein ABC transporter 1, mitochondrial	ABC1
chr9		10,832,291	T	C	5.056321962	JreChr09G10082	mediator of RNA polymerase II transcription subunit 19a-like	MED19A
chr11		4,300,156	G	A	5.137001792	JreChr11G12219	transcription factor MYB1R1	MYB1R1
chr11		4,300,156	G	A	5.137001792	JreChr11G12220	serine/threonine-protein kinase AtPK2/AtPK19	ATPK2
FI (Fruit index)								
chr4		19,311,132	A	G	5.432765952	JreChr04G10670	plant cysteine oxidase 4	PCO4
chr5		34,682,556	G	A	5.842880644	JreChr05G11833	RHOMBOID-like protein 8	KOM
chr7		26,667,119	C	T	5.028817423	JreChr07G11162	protein ABC transporter 1, mitochondrial	ABC1
chr10		24,949,180	G	A	5.329992146	JreChr10G10940	AP2-like ethylene-responsive transcription factor AIL5	AIL5
chr11		629,619	G	A	6.196447364	JreChr11G12397	omega-hydroxypalmitate O-feruloyl transferase	HHT1
chr11		9,836,964	T	A	5.674913712	JreChr11G12686	LEAF RUST 10 DISEASE-RESISTANCE LOCUS RECEPTOR-LIKE PROTEIN KINASE-like 2.8	LRK10L-2.8
chr11		10,142,656	T	A	5.414365813	JreChr11G10006	receptor-like protein kinase FERONIA	FER
chr15		33,763,070	T	C	5.822111878	JreChr15G11477	heptahelical transmembrane protein 2	HHP2
FR (Filling Rate)								
chr3		33,047,998	C	T	5.2309174	JreChr03G11738	senescence-specific cysteine protease SAG39	SAG39
chr11		10,186,241	G	A	5.155535686	JreChr11G10011	metal tolerance protein 10	MTP10

### Comprehensive genetic basis for shell thickness (ST)

**Fig. 3:**
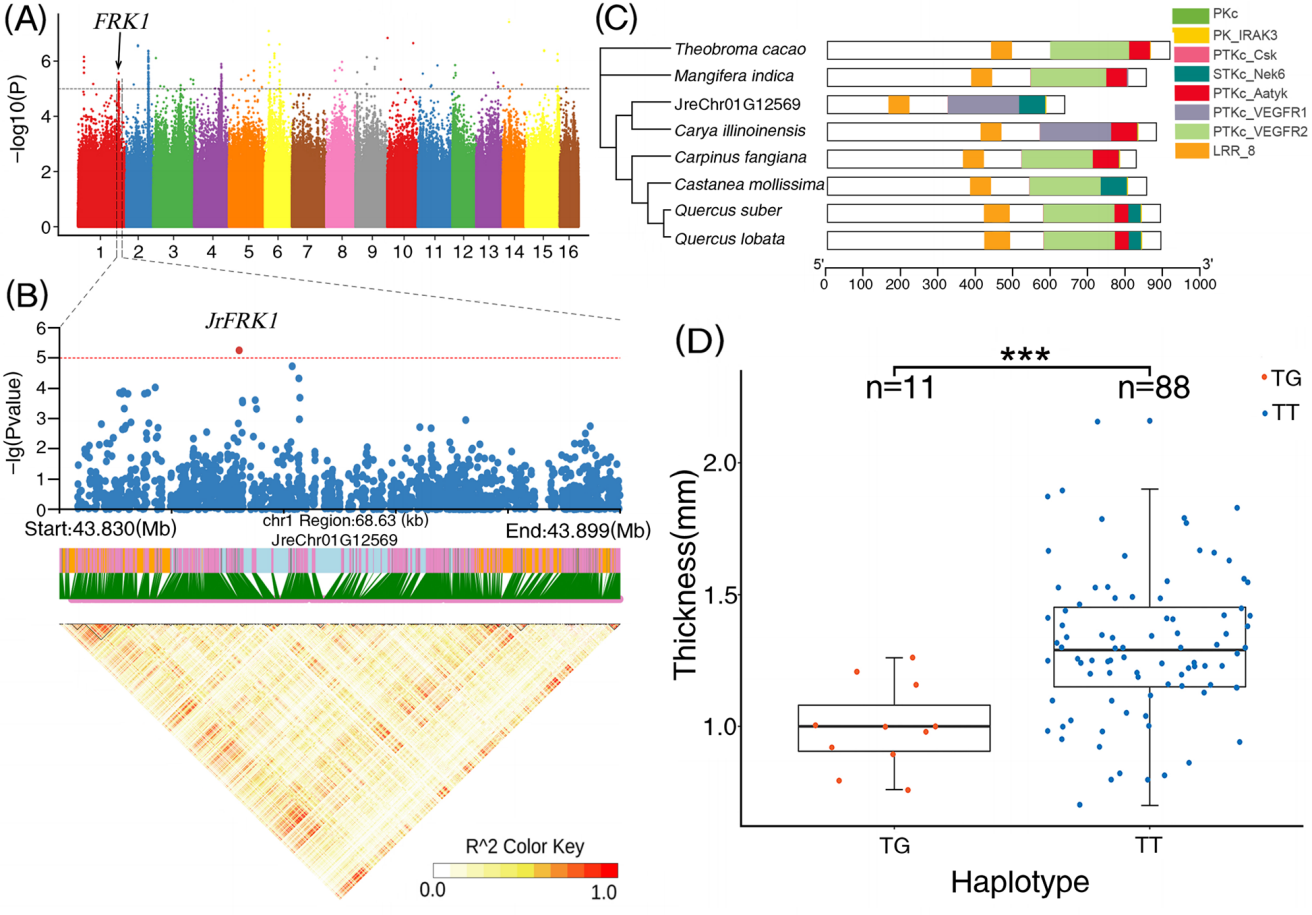
GWAS for shell thickness and in-depth analysis of candidate genes. **A** Manhattan plot shows that SNPs were significantly correlated with walnut shell thickness characteristics. Grey dashed lines represent significance threshold of -log10 (*p*-value); **B** Haplotype map and LDBlock of the *JrFKR1* gene, from 43.83 Mb to 43.90 Mb of chromosome 1; **C** Phylogenetic relationships and conserved domains of FRK1 genes in 11 species; **D** Phenotypic differences between the two *JrFRK1* gene haplotypes

To determine the most important target traits for walnut shell thickness, we conducted a relatively deep investigation to explore its underlying genetic basis. The results of GWAS revealed that the most significant SNPs associated with shell thickness (ST) were located on chromosomes 1, 2, 3, 5, 6, 9, 10, 11, 13, and 14 (Fig. 3A). The significantly higher *p*-value observed, in contrast to that expected in the Q–Q plot, indicated that the potential candidate SNP loci were reliable, highlighting the relatively well-fitted effect of the GWAS model MLM. Initially, 55 candidate LD blocks were calibrated for the target ST traits. The candidate genes were investigated further between these identified LD blocks. According to the annotation results and physical positions (< 5000 bp) between the genes and significance peak signals of the targeted SNP within the LD blocks, 49 genes were finally selected as candidate genes that accounted for the variation in ST.

Furthermore, among these candidate genes, combined with the results of annotation and enrichment analysis, 14 genes with specific functions were selected as the most important candidates associated with the ST (Table 1). Specifically, these candidate genes included “JreChr01G12569” (annotation: FRK1, physical position: chromosome 1, 43,852,395 bp, peak *p*-value: 5.25), “JreChr02G11142” (annotation: ELF3-Like, physical position: chromosome 2, 23,755,810 bp, peak *p*-value: 5.17), “JreChr02G11216” (annotation: IOS1, physical position: chromosome 2, 24,412,382 bp, peak *p*-value: 5.75), “JreChr03G11591” (annotation: STY13, physical position: chromosome 3, 3,094,759 bp; peak *p*-value: 6.11), “JreChr03G11591” (annotation: MBF1, physical position: chromosome 3, 29,892,251 bp; peak *p*-value: 5.13), “JreChr05G10768” (annotation: SRD2, physical position: chromosome 5, 20,620,130 bp; peak *p*-value: 5.48), “JreChr05G11239” (annotation: TMK1, physical position: chromosome 5, 26,729,080 bp; peak *p*-value: 5.66), “JreChr06G10266” (annotation: CYP86B1, physical position: chromosome 6, 15,911,060 bp; peak *p*-value: 6.61), “JreChr09G10847” (annotation: CYP94A1, physical position: chromosome 9, 2,068,541 bp; peak *p*-value: 5.60), “JreChr10G11611” (annotation: PXC1, physical position: chromosome 10, 349,801 bp; peak *p*-value: 6.84), “JreChr11G10209” (annotation: NCED1, physical position: chromosome 11, 13,887,811 bp; peak *p*-value: 5.07), “JreChr11G10535” (annotation: CYP707A2, physical position: chromosome 11, 21,239,109 bp; peak *p*-value: 5.85), “JreChr13G10891” (annotation: GAMYB, physical position: chromosome 13, 23,631,873 bp; peak *p*-value: 5.23), and “JreChr14G11517” (annotation: IQD14, physical position: chromosome 14, 685,568 bp; peak *p*-value: 5.10). Among these, CYP86B1 and CYP707A2 were found to belong to the cytochrome P450 family, which have been reported to regulate plant growth and development. Furthermore, GAMYB (belonging to the MYB family) regulates the traits related to fruit shape, plant resistance, and sex determination.

**Fig. 4:**
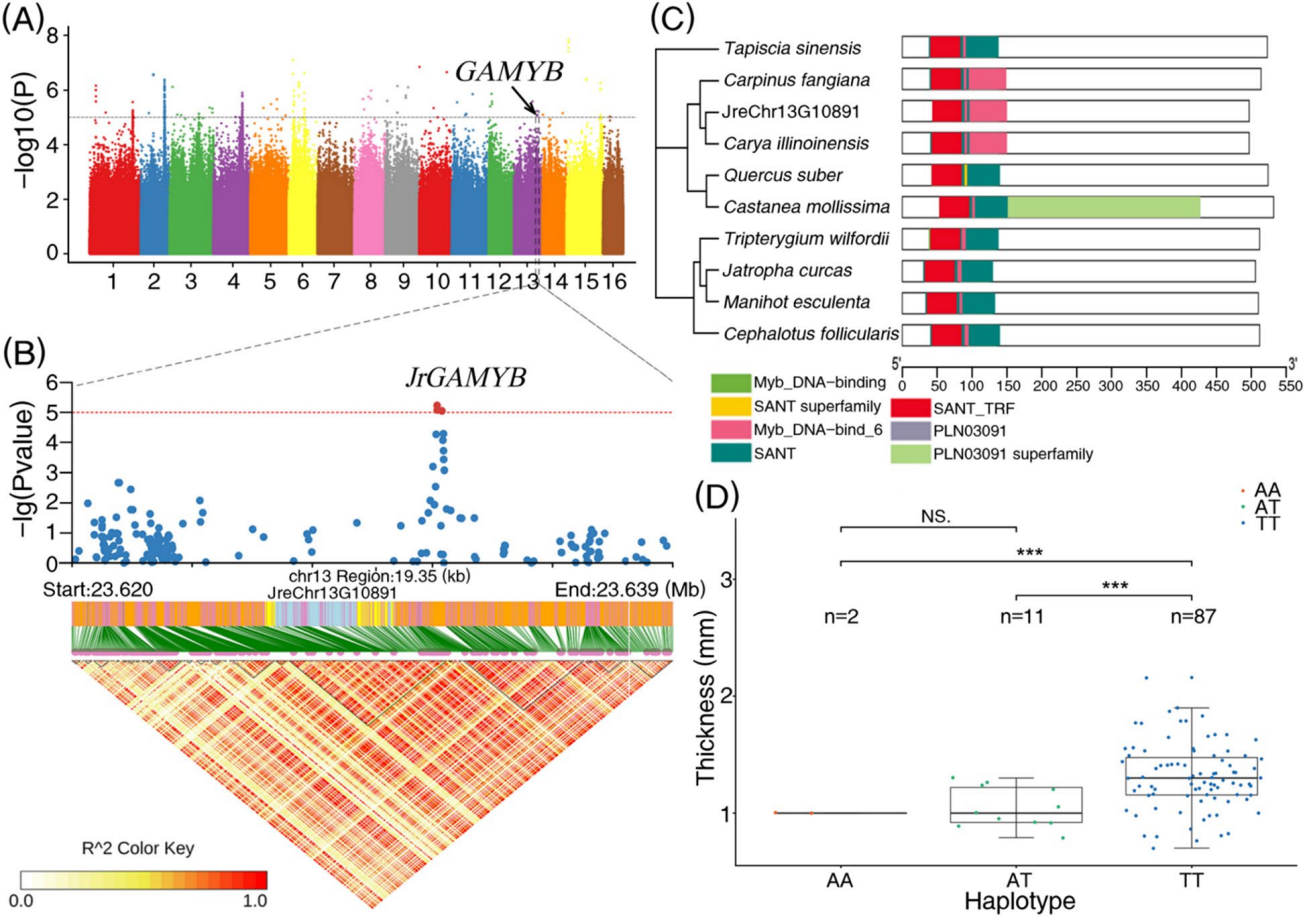
GWAS for shell thickness and in-depth analysis of candidate genes. **A** Manhattan plot shows SNPs significantly associated with walnut shell thickness characteristics. Grey dashed lines represent significance threshold of -log10 (*p*-value); **B** Haplotype map and LDBlock of the *JrGAMYB* gene, from 23.62 Mb to 23.64 Mb of chromosome 13; **C** Phylogenetic relationships and conserved domains of *GAMYB* genes in 11 species; **D** Phenotypic differences between the three *JrGAMYB* gene haplotypes

Furthermore, the JreChr01G12569 gene (FRK1, ranging from 43,843,980 bp to 43,881,173 bp on chromosome 1) with a significant SNP signal (peak *p*-value = 5.25) was considered to be one of the important candidate genes associated with walnut shell thickness (Fig. 3A,B). Phylogenic analysis showed that *JrFRK1* was closely related to FRK in *Carya illinoinensis* among the 10 orthologous genes in this study (Fig. 3C). The gene structure was relatively complex. The LRR_8 domain was commonly shared across all species, whereas, when compared to others, both *J. regia* and *C. illinoinensis* gained the unique PTKc_VEGFR1 domain and lost the PTKc_VEGFR2 domain. A pair of T/G and T/T haplotypes for *JrFRK1* was detected in the studied accessions, among which 11 individuals contained the TG haplotype, while 88 individuals contained the TT haplotype (Fig. 3D). The walnut shell thickness was significantly different between the two haplotype groups, where the TT group was higher than the TG group. Similarly, another gene (JreChr13G10891), located on chromosome 13 (annotated as GAMYB and belonging to the MYB family) and regulating the trait related to fruit shape, was also identified as strongly associated with shell thickness (Fig. 4A,B). This gene was enriched in three KEGG pathways: “BRITE hierarchies”, “protein families: genetic information processing”, and “transcription factors”. JreChr13G10891 (*JrGAMYB*), consisting of five domains including Myb_DNA-binding, SANT superfamily, Myb_DNA-bind_6, SANT, and SANT_TRF, was also gathered into the same clade with homologous genes in *C. illinoinensis* of the phylogenetic tree (Fig. 4C). Three haplotype categories (AA/AT/TT) for *JrGAMYB* were discovered among the studied individuals (Fig. 4D). The number of individuals with the TT genotype was the largest, where the corresponding shell thickness was significantly higher than that of the other two genotypes (two individuals for AA and 11 individuals for AT).

**Fig. 5:**
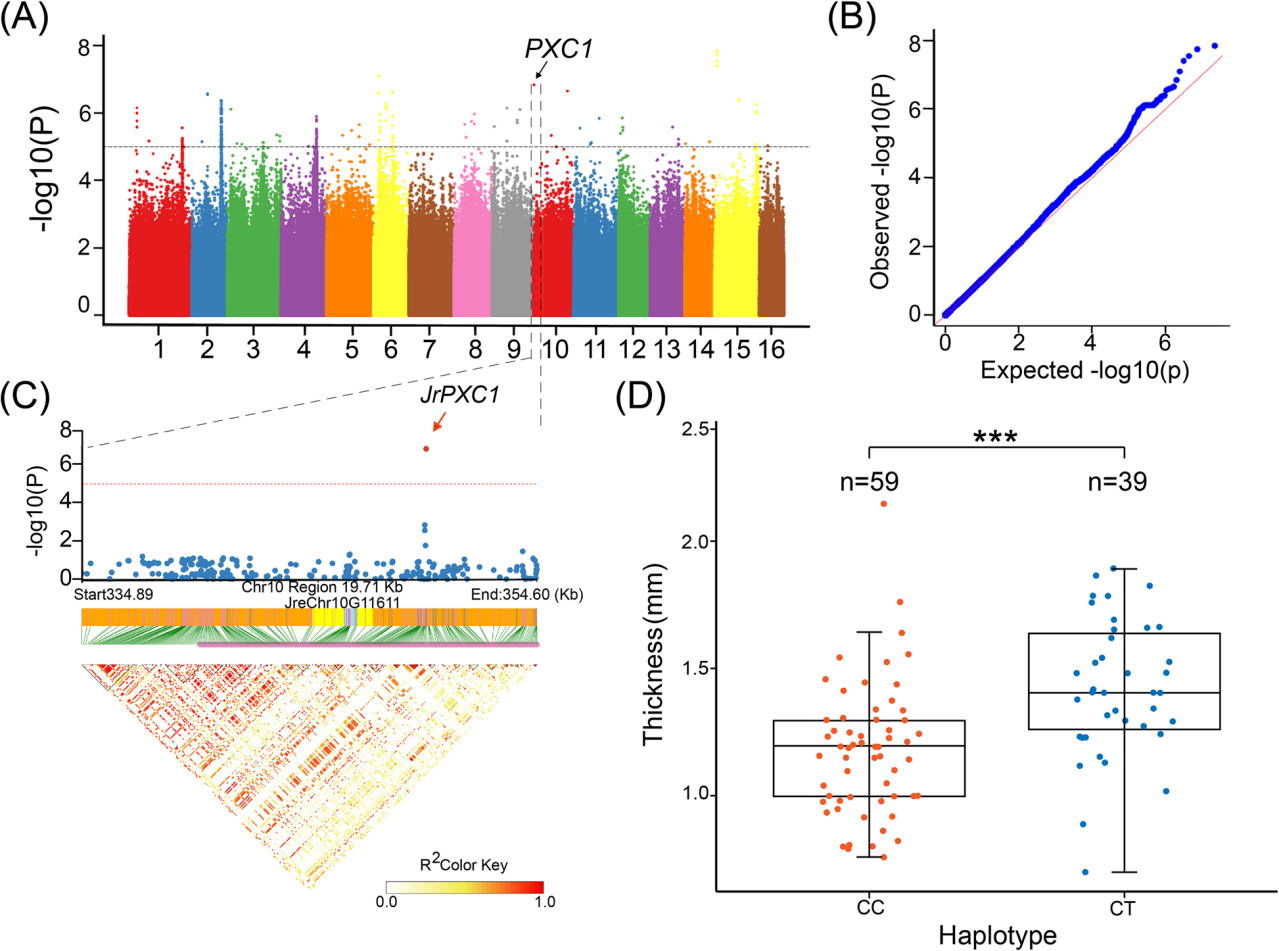
GWAS for shell thickness and candidate gene analysis. **A** Manhattan and **B** Q-Q plots show that SNPs were significantly correlated with walnut shell thickness characteristics. Grey dashed lines represent the significance threshold of -log10 (*p*-value) = 5, black arrows indicate *JrPXC1* (**C**). Haplotype map and LDBlock of the *JrPXC1* gene, from 334.89 kb to 354.60 kb of chromosome 10; **D** Phenotypic differences between the two *JrPXC1* gene haplotypes

**Fig. 6:**
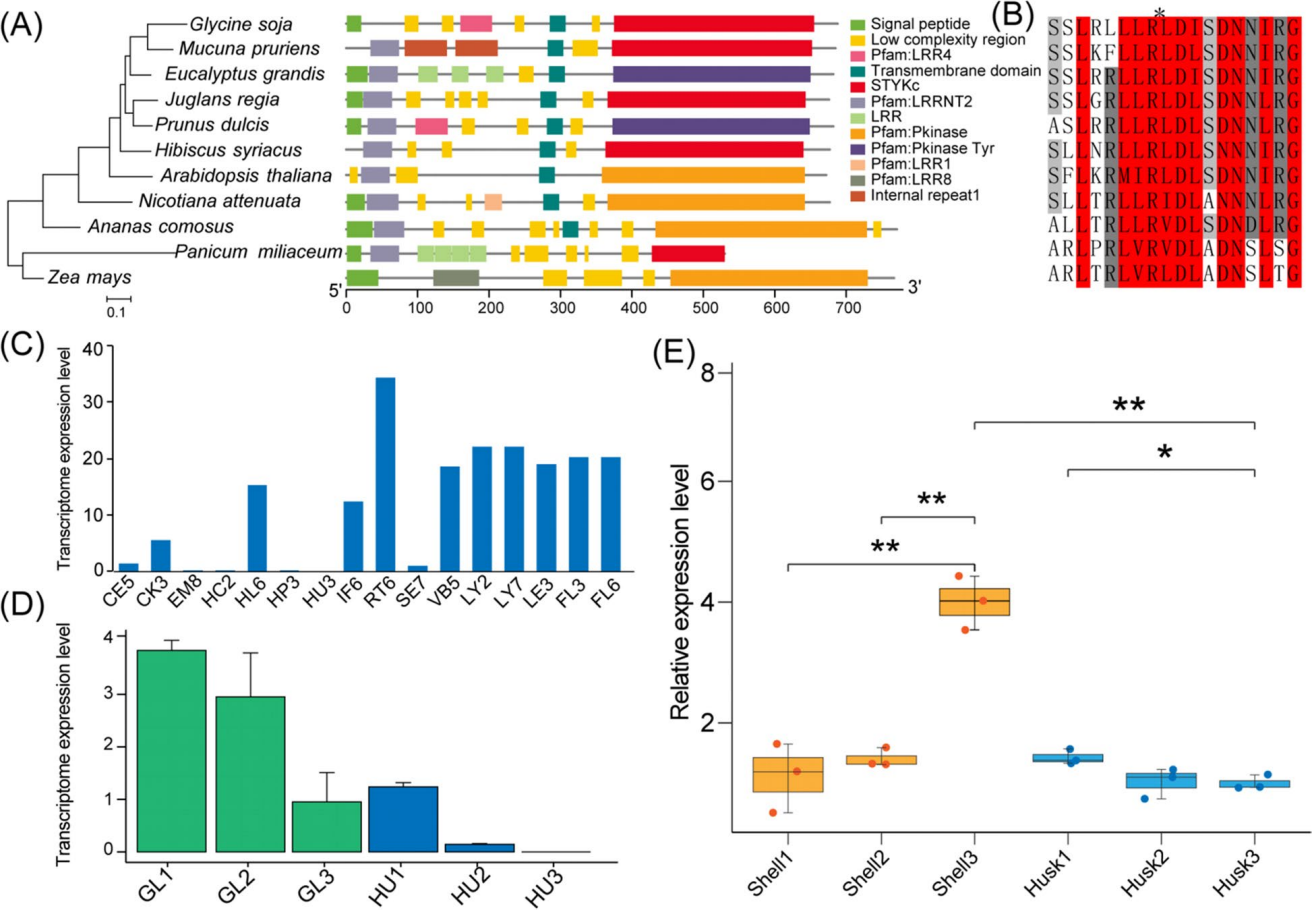
Phylogeny and expression profiles of *JrPXC1*. **A** Phylogenetic relationships and conserved domains of 11 species of PXC1 genes. **B** Protein sequences of PXC1 genes. **C** Expression of *JrPXC1* gene in 17 different tissues. X-axis represents the different tissues of the walnut: CE5 (callus exterior); CK3 (catkins); EM8 (embryo); FL3 (pistillate flower); FL6 (pistillate flower); HC2 (hull cortex); HL6 (hull immature); HP3 (hull peel); HU3 (hull immature); IF6 (fruit immature); LE5 (leaves); LY2 (leaf young); LY7 (leaf young); RT6 (root); SE7 (somatic embryo) and VB5 (vegetative bud). **D** Expression of *JrPXC1* gene in the husk and leaves at four different stages of fruit development. GL indicates walnut green leaves and HU indicates walnut husks. Each of the three biological replicates represents a development period, for a total of four periods. **E** Results of QRT-PCR for *JrPXC1* at three developmental stages of the shell and husk. Samples are from walnut fruit of different periods, * indicates *p* < 0.05, ** represents *p* < 0.01

Lastly, an LD block on chromosome 10 (334.89 kb to 354.60 kb (peak *p*-value = 6.84)) (Fig. 5A,B) contained the gene JreChr10G11611 (*JrPXC1*), which encodes a leucine-rich repeat protein kinase, which was significantly correlated with the walnut shell thickness (Fig. 5C). There was a significant difference in the number of individuals of the two haplotypes (CC/CT) (Fig. 5D). *JrPXC1* was enriched in the GO term “cell periphery and membrane”. It has been reported that PXC1 is involved in secondary cell-wall formation and is responsible for regulating the phenotype. The phylogenetic tree was reconstructed to explore the evolutionary relationship among PXC1 genes in 11 species. The results showed that *JrPXC1* was intimately related to *Prunus dulcis*. All PXC1 genes were found to contain at least one conserved “low-complexity region” domain. Meanwhile, PXC1 genes contained one transmembrane domain, except for *Panicum miliaceum* and *Zea mays* (Fig. 6A). Furthermore, *JrPXC1* possessed five low-complexity regions and shared the conserved domain LRR8 with eight other species (Fig. 6A). Sequence alignment showed that the PXC1 gene was relatively conserved (Figs. 6B and S9).

To further investigate *JrPXC1*, its transcriptome profiles in 17 walnut tissues were analyzed (Fig. 6C). The results revealed that the gene was primarily expressed in roots, leaves, flowers, and vegetative buds. However, this gene was negligibly expressed in the embryo and callus exterior. Furthermore, *JrPXC1* was abundantly expressed at the early stage of husk development (Fig. 6D). When the fruit entered the ripening stage (when the husk ceased to develop), *JrPXC1* was barely expressed. Compared to fruit development, *JrPXC1* exhibited distinct expression patterns during leaf development. During the middle and late stages, despite the declining expression of *JrPXC1* compared with the early stage of leaf development, a continuous relatively high expression of *JrPXC1* was also detected and correlated with cellulose synthesis in the cell wall.

Next, we performed a qPCR-based analysis of the *JrPXC1* gene at different developmental stages of the walnut shell and husk (Fig. 6E; Supplementary Table S4) to further verify its effects. Overall, with shell development, the expression of *JrPXC1* increased significantly, while it was opposite in the husk. The *JrPXC1*expression level in the later stage shell was significantly different from that during the early and middle stages. With shell evolution, the expression of *JrPXC1* increased dramatically, which indicated its important role in regulating shell formation. However, the *JrPXC1* expression level in the early stage of husk development was significantly higher than that in the later stage, presenting a distinct expression pattern compared with shell development.

## Discussion

Walnut production in China has experienced a long history, and China is the leading country in walnut commercialization worldwide (3.63 million tons), followed by the USA and Iran. Due to the climatic diversity and geographical differences in China, there are wide differences in yield, quality, and protection against abiotic and biotic stresses among different walnut varieties [5]. The pressures of environmental degradation and climate change, particularly through drought, salt, and spring frost, are gradually reducing walnut yields. Thus, to face these challenges, native genotypes with robust phenotypic traits adapted to the environment need to be explored and preserved for future development of improved scion and rootstock varieties [47]. This study represents a further achievement in a long-term project for the promotion of molecular breeding in China, as we present a new milestone in walnut genetics to further inspire the introduction of genomics-assisted breeding in tree nut crops.

### Trait variations and correlations

Complex quantitative fruit-related traits are impacted by a variety of physiological and environmental factors that vary widely between walnut genotypes. Genetic and agronomic improvements have significantly increased plant yield potentials. Therefore, it is critical to genetically establish correlations between these traits [29]. Indeed, early walnut breeding studies, as well as those for most perennial woody plants, also proceeded by exploring variations and correlations between interesting phenotypes [7]. Morphological studies can greatly facilitate genetic enhancements by employing selected natural seedling populations [48]. Several phenological traits encompassing lateral bearing [6], dichogamy types [48], bud break [49], and spring frost tolerance [50] were previously adopted for the generation of promising genotypes. Specifically, when focusing on fruit-related traits, there were no statistical differences between protogynous and protandrous nut cultivar characteristics with average yields, while correlations were found between kernel weights and flowering types [8].

Conversely, selected natural walnut populations must possess a sufficiently high genetic diversity, as selecting the correct populations is critical for successful association mapping. In this study, a relatively high phenotypic variation was observed in a number of specific fruit-related traits such as SW, KW, and ST (Table S2), with the CV (coefficient of variation) value corresponding to 27.20, 29.27, and 22.17%, respectively, reflecting variable phenotypic diversity in these studied traits. This was consistent with a recent associative study using a genetic panel with 95 accessions, where the CV value ranged from 8.30 to 46.88%, which corresponded to the NuWi (nut width) and EKeNu (ease of kernel removal from nuts) [51]. The PCA distribution pattern further confirmed high-level diversity for these studied traits (Fig. S1). The cluster analysis of the phenotypic traits among these studied accessions did not form an obvious clade when compared to the molecular phylogenetic analysis (Fig. S6), also indicating the high variability and complexity of these phenotypic traits. China is one of the walnut diversity centers, containing various cultivars and varietals which have experienced long-term domestication. A previous study also indicated that morphological characteristics cannot precisely estimate walnut genetic relationships due to the considerable variability in China [52]. On the other hand, compared to the qualitative traits, the quantitative traits with polygenic control were more sensitive to local environmental conditions [6]. Previous studies also found that leaf budbursts and flowering habits in walnut were significantly influenced by the environment [53], as well as the pellicle color [8] and water-use efficiencies [54]. For this study, the sampling location Guizhou Province was situated in a low-latitude subtropical region on the Yunnan–Guizhou Plateau in China, which is home to a humid subtropical monsoon climate. In this region, the complex terrain combined with a dramatically variable climate over the last 30 years has profoundly impacted both phenotypic and genetic plant diversity [55].

In addition to significant variations in phenotype, correlations also existed between these studied accessions (Fig. 1). Notably, the percentage of FC was significantly negatively related to PC, which inferred the incompatibility of the nutritional composition in walnut fruits, with a similar phenomenon appearing in other crops [56]. Moreover, as anticipated, a positive correlation was found between the phenotypes related to fruit size (LD, SD, and CD) and SW and KW (Fig. 1). A previous association map of nut-related traits in Persian walnut populations from Iran (using an Axiom *J. regia* 700 K SNP array) also revealed a significant positive correlation between these traits [51]. This result was encouraging for breeding since the selection of genotypes with large abundant fruit (LD, SD, and CD) also targets fruit and kernel weights, thus resulting in highly productive plants. Meanwhile, the high level of correlations between these phenotypes suggested a potential linkage of the genes that controlled them in the genome, which provided alternative indicators for genetic mapping via the accession panel.

### Genetic structure and linkage disequilibrium

Genetic structure analysis using different approaches (NJ tree, PCA, and ADMIXTURE) classified our panel into two main groups (Figs. 2, S4, and S5). Although the sampling range of our study was relatively narrow (Guizhou Province, China), the accessions under study were significantly genetically differentiated.

The extent of LD in the genome is one of the most important factors that influences the possibility of LD-based association mapping [57]. Earlier studies proposed that there exist a very different range of LD extensions for various crops and chromosomal regions [58]. As previous studies have indicated, the causes of LD principally include mutation, selection, migration, genetic drift, population bottlenecks, and admixtures [59]. The LD decay (*r*^2^ = 0.2, 3.8 kb and 7.1 kb) observed in our sample collection was most likely due to the open-pollinated origin of our materials, as well as the high levels of genomic recombination in local walnut populations, comparable to those found in other outcrossing woody crops such as grapevine (*Vitis vinifera* L.; 10 kb) (Fig. 2D) [60]. Recent studies focused on other *J. regia* accession GWAS panels also observed a similar LD decay pattern [54].

As a center of diversity and germplasm resources, China has prolonged experience with walnut domestication [52]. A previous study pointed out that the genetic structure and distribution of walnut are profoundly influenced by anthropogenic cultivar selection and the mediated dispersal of selected genotypes [61]. The patterns of genetic diversity and structure we observed for walnut in China were likely a consequence of the complex interactions of evolutionary forces, such as adaptation/ecotype differentiation and human distribution [61]. Consequently, strong artificial selection may be the driving force behind significant linkages. Overall, the LD decay patterns indicated the great potential of Chinese sample collections for GWAS, as previously demonstrated for nut quality-related traits [51]. Nevertheless, to some extent, the relatively small sample size for our association panel limited the power of our GWAS.

### Genotype and phenotype associations

As further proof of the value of our sample collection, association mapping was performed for fruit-related traits, which identified marker–trait associations for the 10 traits under study. Fruit-related characteristics are important when selecting and developing new varieties. Traits such as kernel color, nut size, kernel percentage, and shell thickness are essential criteria for walnut marketing [62], which have been extensively studied for other crops [63]. Therefore, deciphering their genetic control is fundamental toward assisting walnut breeders in the rapid development and introduction of improved cultivars.

For this study, significant SNPs were initially identified via the GWAS panel for the studied traits (Table S6). Combined with LD block analysis, 1212 SNPs within 438 LD blocks were selected for surveying underlying candidate genes that contributed to phenotype variations. Furthermore, through gene function annotation and GO and KEGG enrichment analysis, 61 candidate genes were finally identified accounting for these studied traits (Table 1).

For the LD (longitudinal diameter), the candidate IAA16 (auxin indole-3-acetic acid 16) gene located on chromosome 7 (Table 1) was one of the transcriptional repression factor auxin/indoleacetic acid (AUX/IAA) proteins. Auxin regulates many aspects of plant growth and development [64]. A resistance endowing IAA16 mutation was recently elucidated, which leads to significant vegetative growth defects and impaired competitiveness in *Bassia scoparia* [65]. Interestingly, this gene was also associated with the KW (kernel weight) (Table 1), indicating the important role of IAA16 in responding to the development of walnut fruit.

The candidate FER (FERONIA, a receptor-like kinase) gene on chromosome 11 was found to be involved in LD, SD, and FI (Table 1). According to a previous study, FER was shown to function in several growth-regulatory pathways, including root hair elongation regulated by auxin and cell growth induced by other hormones [66]. Yu et al. reported that FER was a positive regulator of auxin-promoted growth, to suppress the abscisic acid (ABA) response through the activation of a negative ABI2 regulator of ABA signaling [67]. In another GWAS panel for walnut [53], the FER gene was identified as temporally mediating leaf budburst, reflecting its essential role in the regulation of walnut development.

Both candidate ERG3 and KAS3A genes were correlated with three traits (ERG3 for LD, SD, and SW) (KAS3A for LD, CD, and SW). ERG3 (elicitor-responsive protein 3) encodes a small C2-domain protein, which is a common Ca^2+^-dependent lipid-binding motif that is abundantly present in membrane-associated proteins involved in both signaling and membrane trafficking [68]. Most small C2-domain proteins have been shown to be involved in plant defenses or stress responses [69]. Furthermore, KAS3A (3-oxoacyl-[acyl-carrier-protein] synthase 3 A) is involved in the regulation of aliphatic acid biosynthesis by catalyzing the condensation of acetyl-COA with a malonyl-acyl carrier protein in dissociated (type 11) fatty-acid synthase systems. A recent study of *Gossypium arboretum* for 243 accessions revealed that the KAS3A gene was significantly correlated with the aliphatic acid content [70]. The fat content of a walnut kernel ranges from 60 to 70%, with unsaturated fatty acids accounting for ~ 90%. Previous studies confirmed that the health benefits of walnut are primarily due to the walnut kernel, which is rich in unsaturated fatty acids (e.g., linoleic acid, α-linolenic acid, and oleic acid) that have potent effects for lowering cholesterol, as well as preventing and alleviating cardiocerebrovascular disease, diabetes, and obesity [71].

Moreover, the JreChr03G12405 gene located on chromosome 3 was annotated as “zinc finger BED domain-containing protein DAYSLEEPER-like”, containing four conserved domains: “Streccoc_I_II”, “ZnF_BED”, “DUF4413”, and “Dimer_Tnp_hAT”. The gene was significantly related to the SD and KW (Table 1). The BED-type zinc finger and hAT dimerization domains are unique to higher plants (basal angiosperms, as well as grasses (Poaceae) and dicotyledonous plants), related to plant rhythms with important roles in plant growth and development [72].

The kernel and signal weights were significantly correlated and typically controlled by three candidate genes: WAKL8 on chromosome 4, CKP11 on chromosome 5, and CSLG3 on chromosome 11 (Table 1). WAKL8 is a member of the WAK (wall-associated kinase) family, which plays important roles in signal transduction between the cell wall and cytoplasm in plants. In addition to our finding, Li et al. identified 27 WAK/WAKL genes in *J. regia* [73]. Being the only type of receptors involved in cell-wall signaling, WAK genes can directly transfer signals from the extracellular to cytoplasm domains, as well as facilitate plant cell expansion, metal tolerance, resistance against plant diseases, and responses to various plant hormones and abiotic stresses [74]. Furthermore, CKP11 (Ca^+^-dependent protein kinase 11) is involved with dehydration responses and has been identified in various plants [75]. Increased free Ca^2+^ in the cytoplasm may trigger a signal for stomatal closure to reduce further water loss. The expression of CKP genes is rapidly induced by drought and high-salt stress, as well as the plant hormone abscisic acid (ABA) [76]. CSLG3 (cellulose synthase-like protein G3) is involved in the synthesis of cellulose in plants. Cellulose and lignin can be tightly crosslinked to form a hydrophobic network that enhances the mechanical strength of plant cell walls, which makes them more resistant to infection of pathogens [77]. In summary, the genes involved in cell-wall development and reinforcement might be the key factors behind walnut kernel and fruit development.

In addition, MYB1, MYB106, and 4CL2 were identified as being responsive to KW (Table 1). Among all candidates, the MYBs were enriched in the following KEGG pathways: “transcription factors”, “circadian rhythm—plant”, “environmental adaptation”, and “protein families: genetic information processing”. On the other hand, 4CL2 was enriched in “phenylpropanoid biosynthesis”, “ubiquinone and another terpenoid-quinone biosynthesis”, and “metabolism of cofactors and vitamins”. These results indicated that MYB and 4CL are important transcription factors for walnut development and growth, and earlier studies suggested that they play critical roles in the metabolism of plant flavonoids [78]. Walnut kernels are rich in flavonoids with strong biological activities. As important signaling molecules and allelochemicals during plant growth and development processes, flavonoids play essential roles in regulating the physiological metabolism of plants [79]. Additionally, MYB transcription factors were found to be associated with cytokinin-regulated cell division, which causes an increase in size and weight of the entire fruit during the early stage of walnut fruit development [8]. MYB35, GAMYB, and MYB1R1 were also significantly associated with CD, ST, and PC, respectively (Table 1).

In terms of the protein content (PC) trait, JreChr03G10302 (ABC1K8), JreChr07G11142 (ABCB21), and JreChr07G11162 (ABC1) loci, coding for the ABC transporter, were considered to be important genes. Marrano et al. found that the ABC transporter was significantly correlated with the leafing date via the 700 K SNP array walnut GWAS panel [8]. ABC transporters have a broad presence in organisms; to date, they are one of the largest and most functional transporter families known, which play important roles in the transport of transmembrane substances in both eukaryotes and prokaryotes [80]. A previous study showed that seven ABC genes were upregulated during fruit development in *Vaccinium corymbosum* [81], confirming the critical role of ABC transporters.

### Genetic basis for walnut shell thickness

The walnut fruit consists of a kernel, pellicle, shell, and husk. The embryo that forms the kernel initially becomes apparent approximately 7 weeks after pollination and increases in size within the husk cavity until late July [8]. During this period, the shell forms and hardens, containing cells that differentiate and lignify. Shell thickness is a key component of shell integrity, which is an important economic trait and an essential criterion for walnut marketing [9].

Walnut shells need to remain intact during harvest and storage (i.e., during tree shaking, transportation, cleaning, and drying) to exclude dirt, insects, moisture, or other contaminants. Shell thickness and suture strength were found to be significantly correlated with broken kernels and insect damage, whereby kernel breakage can result in increased microbial damage and a decreased antioxidant capacity [82]. Researchers in Iran, Turkey, and China have quantified walnut, hazelnut, and macadamia nut suture strength [83] as an essential trait for their industries. Interestingly, the shell thickness was significantly positively correlated to the fruit weight (Fig. 1), reflecting the prominent significance of the shell thickness to walnut yields.

To date, only a few studies have focused on walnut shell thickness. Using the *J. regia* 700 K SNP array, Sideli et al. preliminarily explored candidate genes associated with walnut shell suture strength [9]. In this study, 14 candidate genes associated with walnut shell thickness were identified, integrating the results of GWAS, LD block, gene annotation, and enrichment analysis. Furthermore, three candidate genes, FRK1, PXC1, and GAMYB, were meticulously analyzed. FRK1 (FLG22-induced receptor-like kinase 1) as a plant resistance (R) gene, whose transcription level is regulated by the MAPK (mitogen-activated protein kinase) pathway and downregulated in response to nanoparticles [84], plays an important role in the downstream defense responses of plant pathogens [85]. Sideli et al. also identified a leucine-rich repeat receptor-like protein kinase (PXL1) in response to the shell suture strength, which, as the largest transmembrane receptor kinase subfamily in plants, not only plays a critical role in plant growth and development, but also participates in disease resistance and defense. These results suggested that disease resistance genes might significantly contribute to the development of the walnut shell. As a physical barrier to protect walnut kernels, the shell filters out and obstructs the entry of most pathogens [9]. In this study, two types of haplotypes were observed for the SNP loci associated with FRK1, while the homozygous genotype T/T significantly corresponded to the shell thickness at a much higher level than that of the heterozygous genotype G/T (Fig. 3D).

GAMYB, located on chromosome 13, was considered to be another important candidate gene related to the walnut shell thickness. The MYB gene family was identified as playing important roles contributing to the fruit-related traits in our study (Table 1). GAMYB (gibberellin acid MYB) was enriched in the “transcription factor” and “protein families: genetic information processing” KEGG pathways. As a vital plant hormone, gibberellin plays an important role in various stages of plant growth and development, where its physiological function primarily depends on signal transduction. GAMYB is an MYB transcription factor induced by GA, representing the first positive factor identified in GA signal transduction. The GAMYB protein induces the expression of downstream genes by binding to the promoter of the GA response gene [86]. The results of this study indicated that the GA signal transduction pathway might play an important role in the biosynthesis and development of walnut shell. A total of 85 accessions with the T/T haplotype for GAMYB in this study exhibited a significantly higher shell thickness than the AA and AT haplotypes, implying that the shell thickness is closely mediated by GAMYB (Fig. 4D). However, the exact underlying regulatory mechanisms need to be comprehensively explored in the future.

Lastly, a block on chromosome 10 in the walnut genome, from 334.89 kb to 354.60 kb with a notable SNP JreChr10G11611-PX1 (physical position: 344,875 bp; peak value = 6.84), was significantly correlated with the shell thickness (Fig. 5C). This region corresponded to the *JrPXC1* gene, which also encodes a leucine-rich repeat protein kinase (LRR) involved in secondary cell-wall formation in xylem fibers [87] and responsible for regulating the phenotype in *Arabidopsis thaliana*. A soybean orthologous *PXC1* (GmLRK1) was shown to be involved in the development of the cell-wall architecture, where the mutant of this gene induced a defect in leaf cell elongation [88]. In this study, the phylogenetic results indicated that the *JrPXC1* gene was closer to that in *Prunus salicina* (Fig. 6A). The change from C to T at 349,801 bp on chromosome 10 in *JrPXC1* caused a significant difference in shell thickness (Fig. 5D). According to an earlier study, we speculated that the mutation in the intron of the *JrPXC1* gene may cause abnormal splicing during transcription. This results in changes in the composition and structure of mRNA, in turn impacting the maturation of mRNA and, consequently, the formation of cellulose in the secondary cell wall [88].

The transcriptome profiles and qRT-PCR were used to further explore the distribution patterns and intensity of *JrPXC1* expression. The results revealed that the *JrPXC1* gene was abundantly expressed during secondary cell-wall construction, indicating that it strongly affected the thickness of the walnut shell (Fig. 6D). The distinct expression pattern obtained from qPCR for *JrPXC1* in the shell and husk revealed an unsynchronized development strategy for these two tissues. Specifically, the walnut shell is induced to form rapidly as husk development tends to terminate, which results in different, albeit simultaneous *JrPXC1* expression patterns in various portions of the fruit [89].

## Conclusion

For this study, a set of 101 walnut accessions were employed to evaluate fruit-related traits using a GWAS with a mixed linear model. Utilizing multiple analyses, the candidate *JrFRK1*, *JrGAMYB,* and *JrMYB35* genes, as well as other novel genes, were discovered as correlating to the morphological development of the fruit. Certain candidate genes obtained in this study, such as IAA16 and CKP11, participated in hormone regulation. Most of the candidate genes were associated with the development and disease resistance of fruits, indicating that they had an important influence on fruit growth. Following an in-depth analysis, the candidate *JrPXC1* gene was identified as a likely target gene, which regulated walnut shell thickness. An elucidation of the functionalities of the genes that mediate these critical agricultural traits will deepen our understanding of the mechanisms that facilitate walnut development. This study may potentially assist with the development of improved cultivar qualities and value-added varieties to meet the demands of consumers and industry.

## Supplementary Information

**Additional file 1: Figure S1.** The correlation heatmap of 101 individuals based on 10 traits. The phenotypes from left to right were LD (Longitudinal diameter), CD (Cross diameter), SD (Side diameter), FI (Fruit index), SW (Single weight), NW (Nut weight), FR (Filling rate), ST (Shell thickness), FC (Fat content), PC (Protein content), respectively. **Figure S2.** The distribution of SNPs on 16 chromosomes mapped to reference genome. **Figure S3.** The distribution of InDels on 16 chromosomes mapped to reference genome. **Figure S4.** The population structure of 101 walnut accessions from *K*=2 to *K*=4 by using the software Admixture v1.3. **Figure S5.** Principal component analysis (PCA) of 101 walnut accessions. PCA analysis based on **(A)** PC1 (6.75%) and PC2 (3.65%), **(B)** PC2 (3.65%) and PC3 (2.85%), and **(C)** PC1 (6.75%) and PC3 (2.85%), respectively. **Figure S6.** Cluster analysis on phenotypic traits of 101 walnut accessions. Clustering based on methods of **(A)** Neighbor-joining (NJ) and **(B)** unweighted pair group method with arithmetic mean (UPGMA). **Figure S7.** The Manhattan plot of the 10 phenotypic association results and the corresponding Q-Q plot. The results are calculated by the GEMMA software, and the grey dotted line represents the significant threshold. **(A)**. CD; **(B)**. FC; **(C)**. FI; **(D)**. FR; **(E)**. LD; **(F)**. NW; **(G)**. PC; **(H)**. SD; **(I)**. ST; **(J)**. SW. **Figure S8. (A)** The GO analysis of associated genes in shell thickness. **(B)** The KEGG enrichment analysis of associated genes in shell thickness. **Figure S9.** The protein sequence alignment of the *PXC1* gene homologs in 11 plants.

**Additional file 2: Supplementary Table S1.** Overview of Illumina sequencing data produced and alignment to the reference genome assembly. **Supplementary Table S2.** The statistics of ten phenotypic traits in 101 walnut accessions used in this study. **Supplementary Table S3.** The information and annotation of SNPs detected in 101 walnut accessions. **Supplementary Table S4.** Transcriptome data corresponds to the full name of the organization. **Supplementary Table S5.** Sample information for qRT-PCR expression levels of JrPXC1 gene between walnut shell and husk. **Supplementary Table S6.** The phenotypes with significant SNP information obtained by GWAS.

## Data Availability

The datasets necessary for supporting the results of this article are included in this manuscript and its additional files. The datasets generated and/or analysed during the current study are available in the National Center for Biotechnology Information (NCBI) repository with accession number BioProject PRJNA782855.

## Abbreviations

GWAS: Genome-wide association study
SNPs: Single nucleotide polymorphisms
NGS: Next-generation sequencing technologies
LD: Linkage disequilibrium
WIP: Walnut Improvement Program
LD: Longitudinal diameter
CD: Cross diameter
SD: Side diameter
SW: Single nut weight
KW: Kernel weight
ST: Shell thickness
FC: Fat content
PC: Protein content
FI: Fruit index
FR: Filling rate
NJ tree: Neighbor-joining tree
PCA: Principal component analysis
CV errors: Cross-validation errors
MLM: Mixed linear model
CV: Coefficient of variation
