# Supplementary material for: Genome-wide association analysis of 101 accessions dissects the genetic basis of shell thickness for genetic improvement in Persian walnut (Juglans regia L.)
Source: BMC Plant Biol. 2022 Sep 13;22:436. doi: 10.1186/s12870-022-03824-1 (PMC9469530; doi:10.1186/s12870-022-03824-1)

**Additional File 1**

**Figure S1.** The correlation heatmap of 101 individuals based on 10 traits. The phenotypes from left to right were LD (Longitudinal diameter), CD (Cross diameter), SD (Side diameter), FI (Fruit index), SW (Single weight), NW (Nut weight), FR (Filling rate), ST (Shell thickness), FC (Fat content), PC (Protein content), respectively.


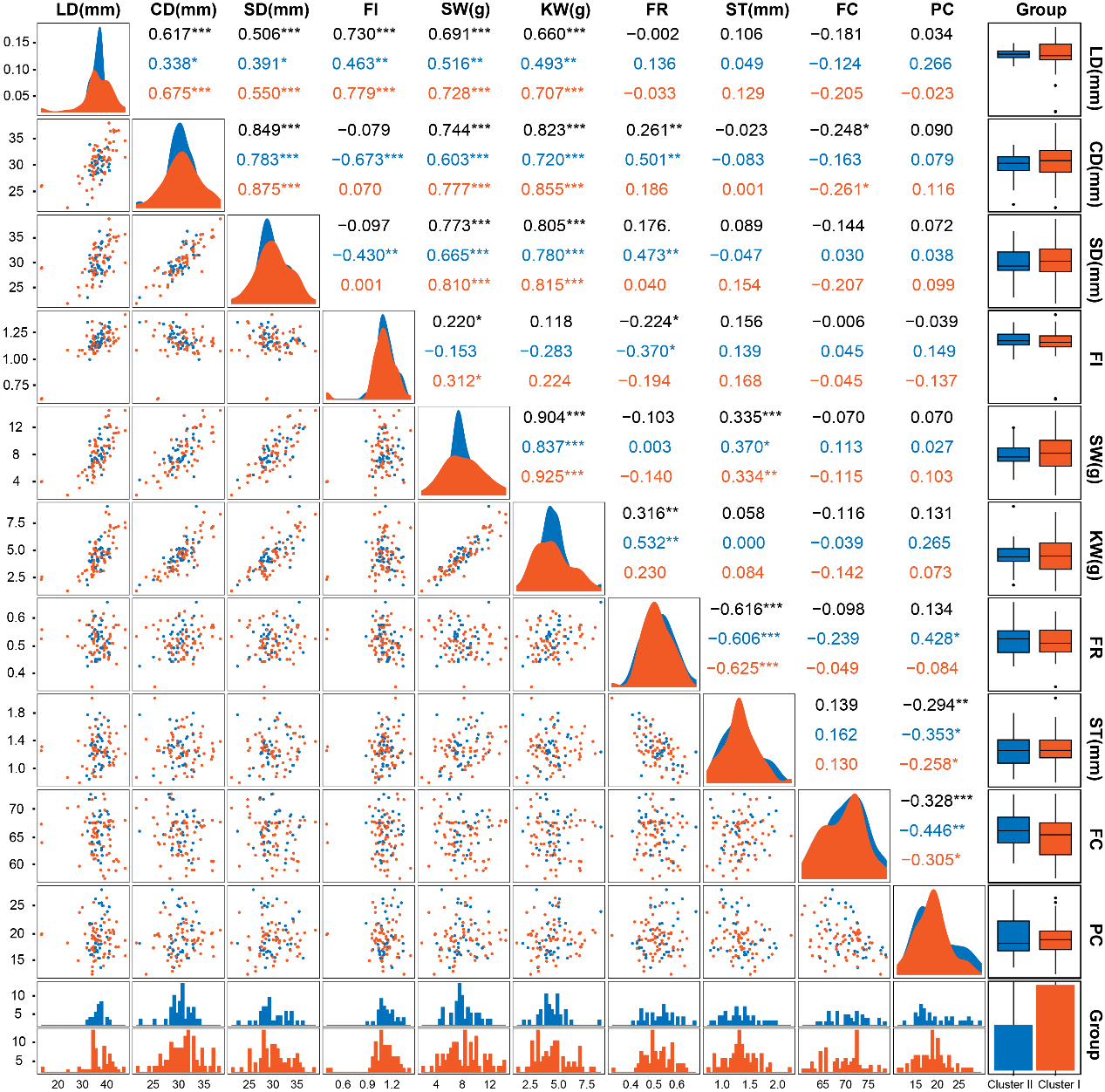


**Figure S2.** The distribution of SNPs on 16 chromosomes mapped to reference genome.


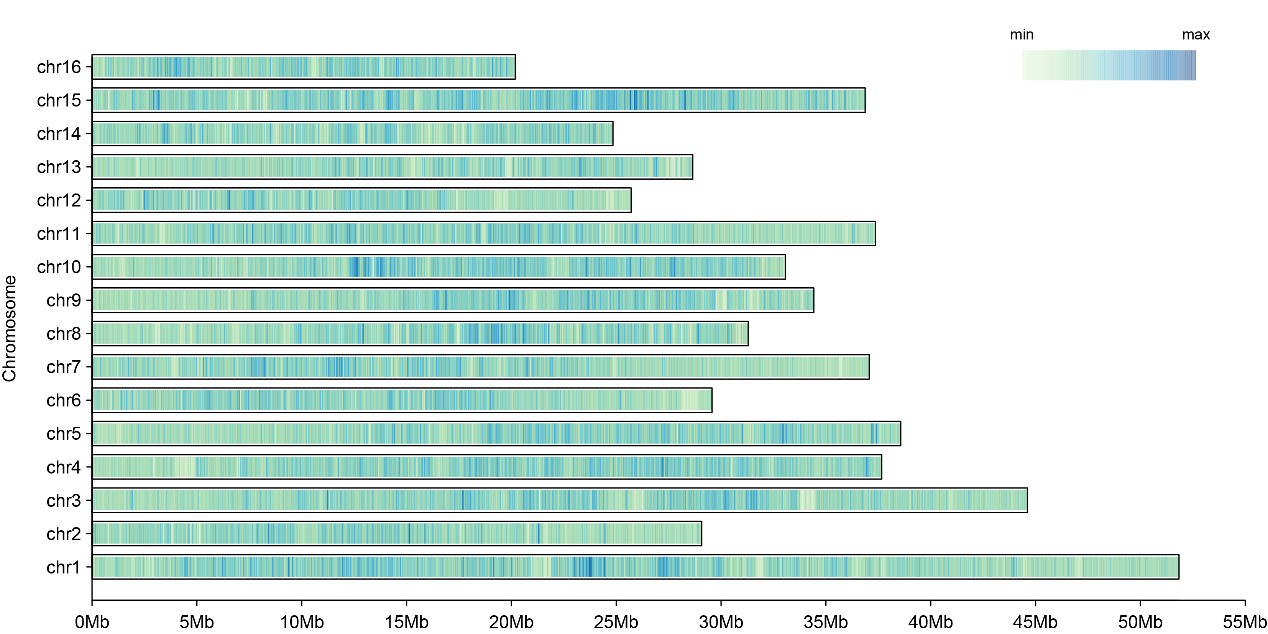


**Figure S3.** The distribution of InDels on 16 chromosomes mapped to reference genome.


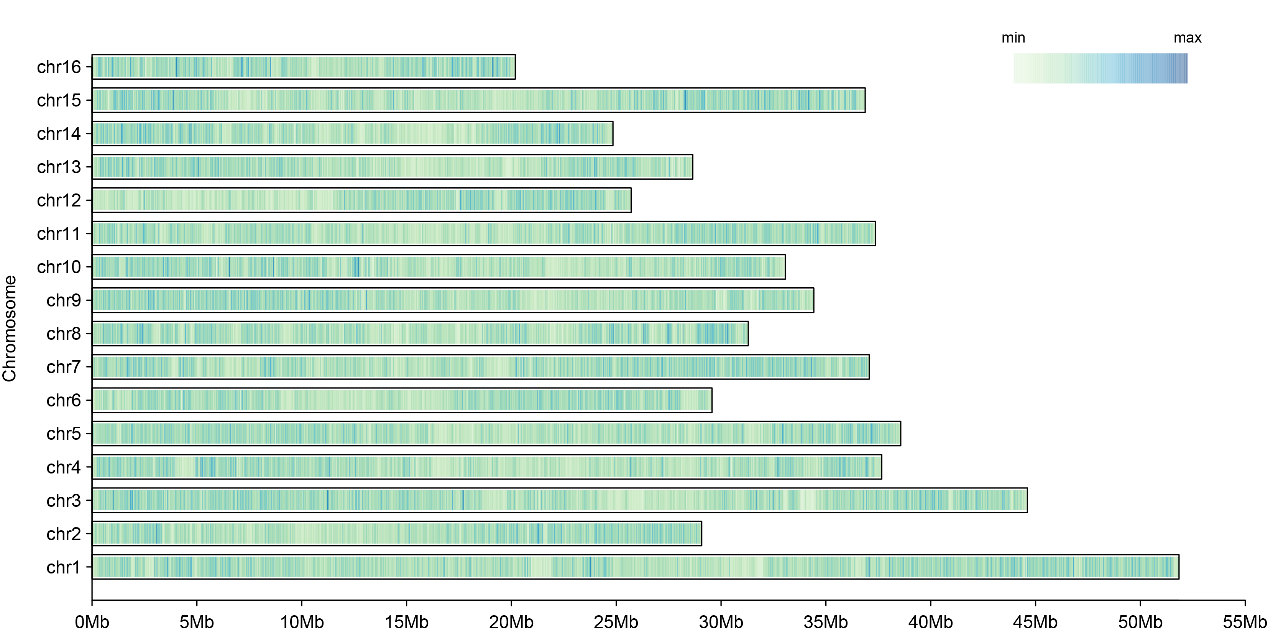


**Figure S4.** The population structure of 101 walnut accessions from *K*=2 to *K*=4 by using the software Admixture v1.3.


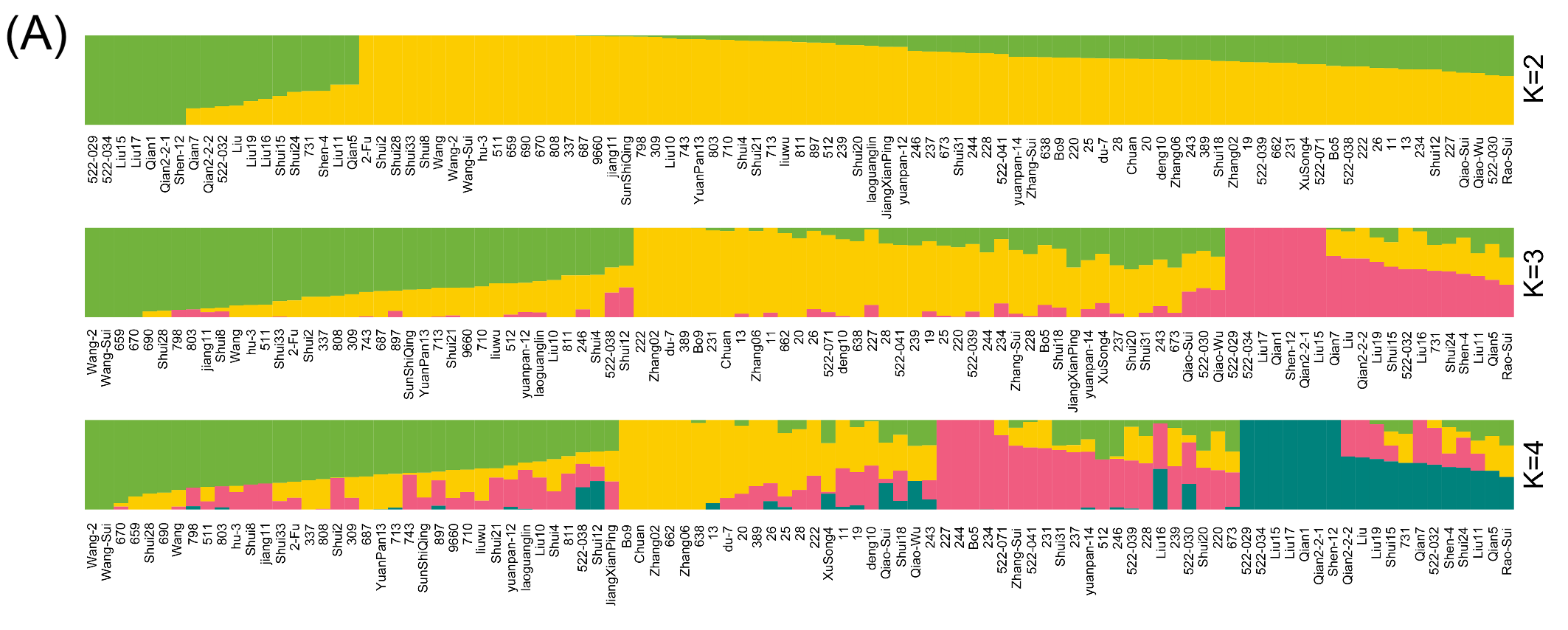


**Figure S5.** Principal component analysis (PCA) of 101 walnut accessions. PCA analysis based on **(A)** PC1 (6.75%) and PC2 (3.65%), **(B)** PC2 (3.65%) and PC3 (2.85%), and **(C)** PC1 (6.75%) and PC3 (2.85%), respectively.


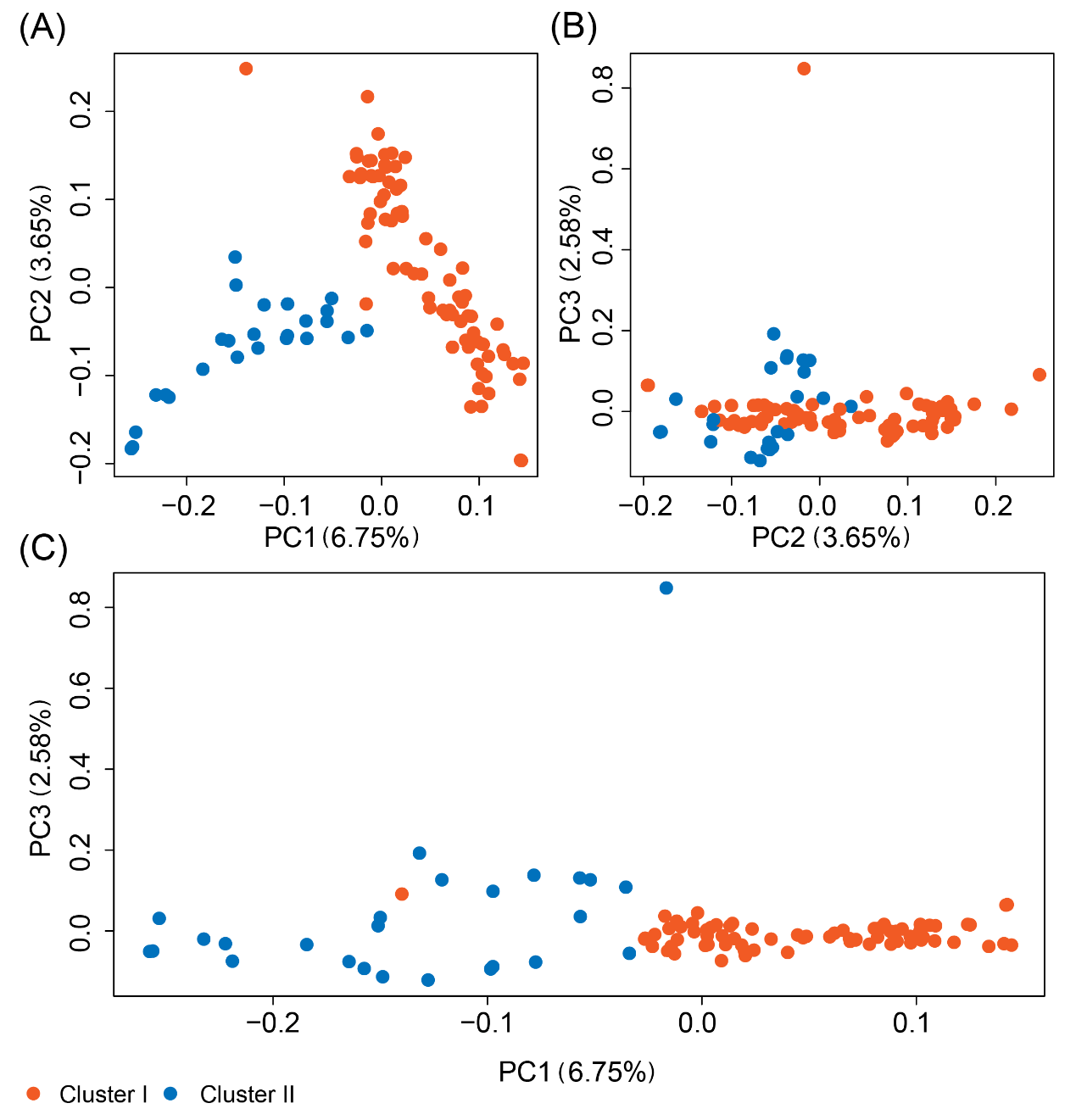


**Figure S6.** Cluster analysis on phenotypic traits of 101 walnut accessions. Clustering based on methods of **(A)** Neighbor-joining (NJ) and **(B)** unweighted pair group method with arithmetic mean (UPGMA).


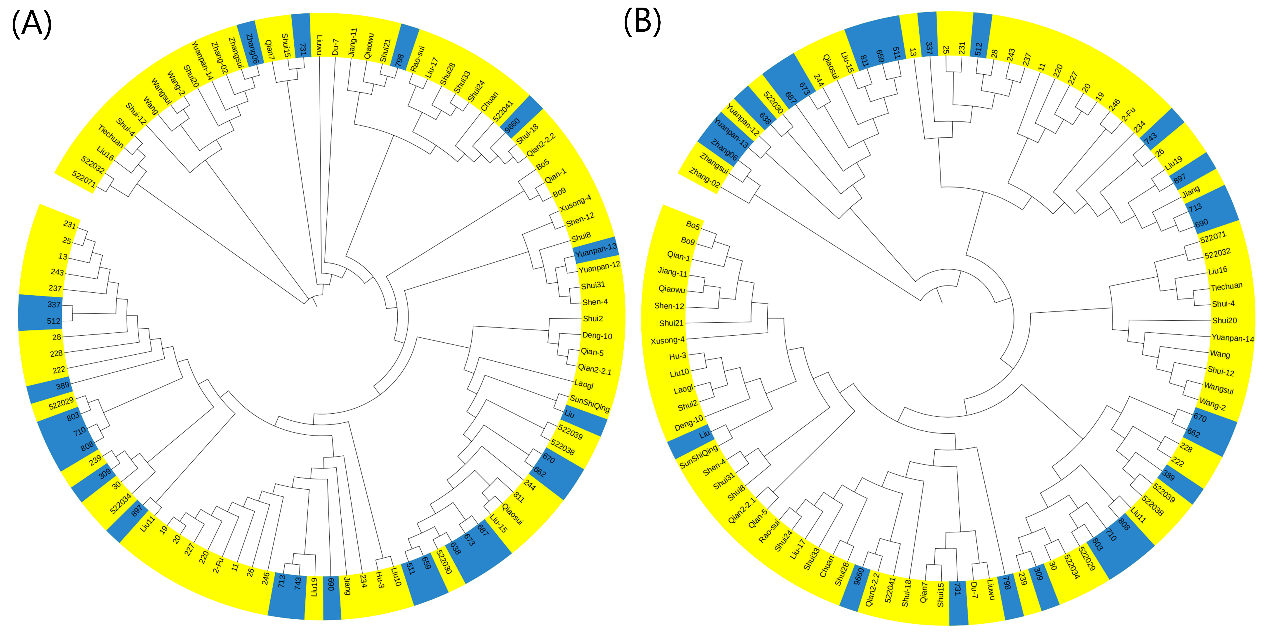


**Figure S7.** The Manhattan plot of the 10 phenotypic association results and the corresponding Q-Q plot. The results are calculated by the GEMMA software, and the grey dotted line represents the significant threshold. **(A)**. CD; **(B)**. FC; **(C)**. FI; **(D)**. FR; **(E)**. LD; **(F)**. NW; **(G)**. PC; **(H)**. SD; **(I)**. ST; **(J)**. SW.


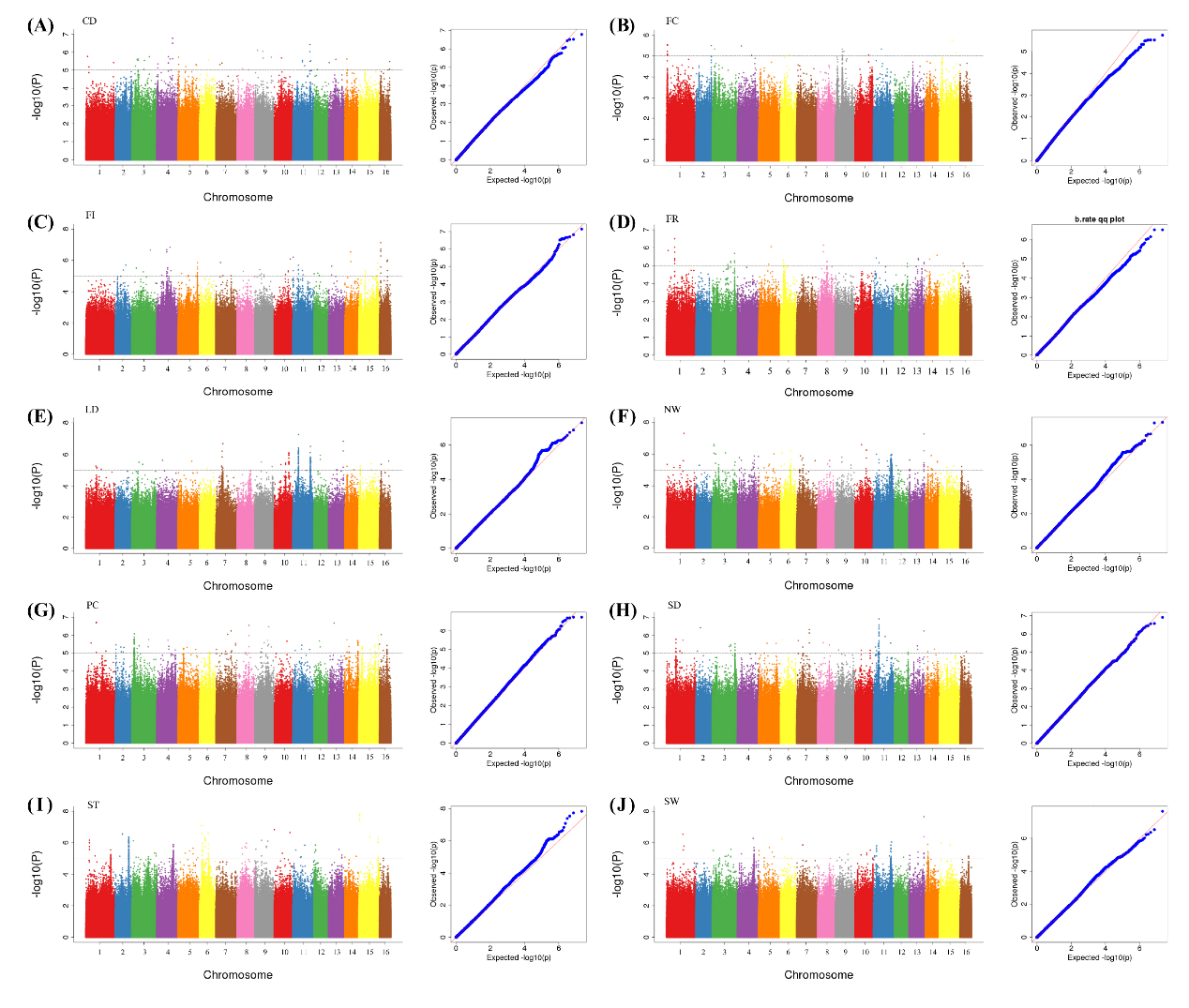


**Figure S8. (A)** The GO analysis of associated genes in shell thickness. **(B)** The KEGG enrichment analysis of associated genes in shell thickness.


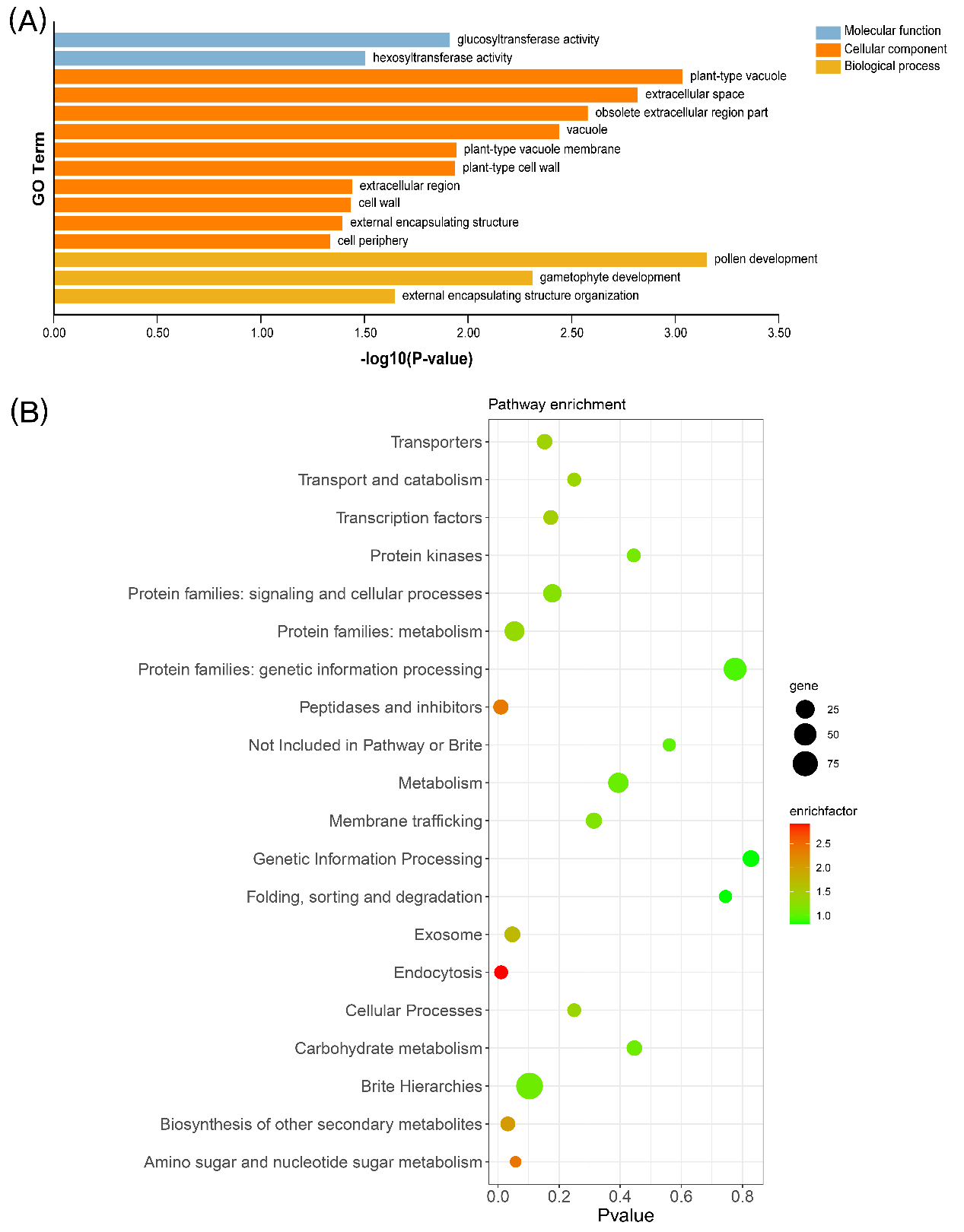


**Figure S****9.** The protein sequence alignment of the PXC1 gene homologs in 11 plants.


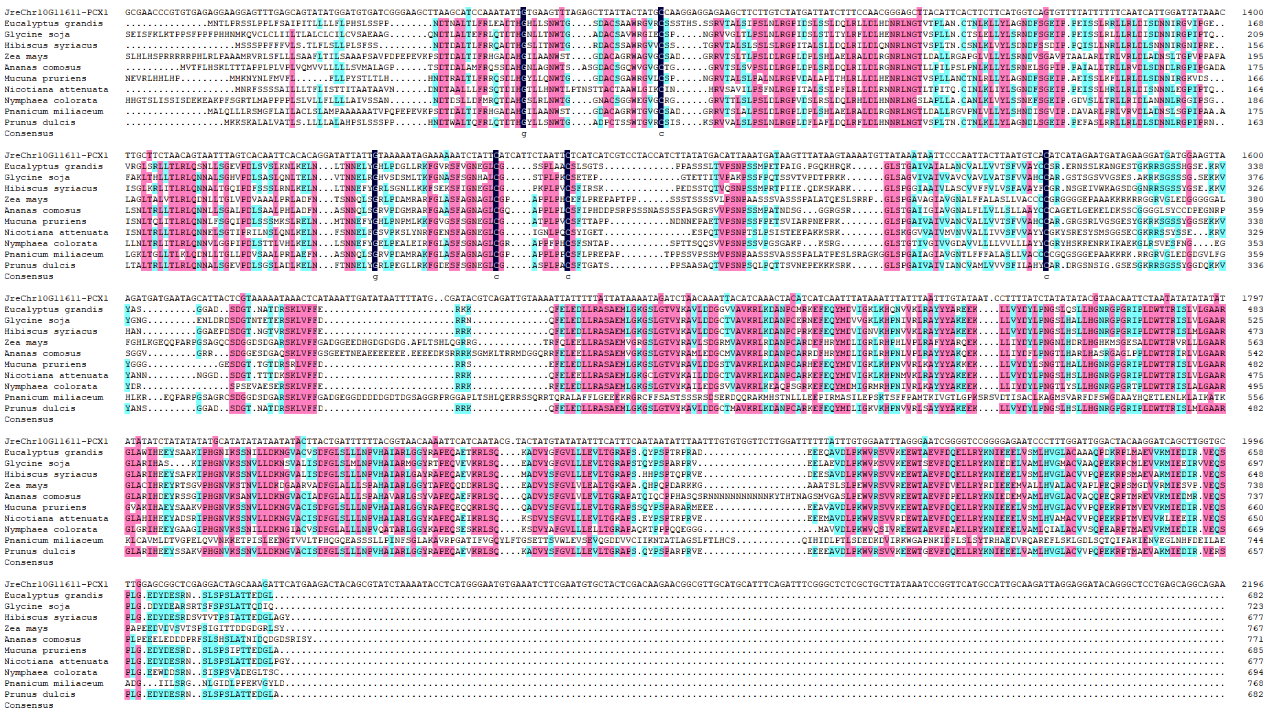

Supplement: Supplementary file 1 — Additional file 1: Figure S1. The correlation heatmap of 101 individuals based on 10 traits. The phenotypes from left to right were LD (Longitudinal diameter), CD (Cross diameter), SD (Side diameter), FI (Fruit index), SW (Single weight), NW (Nut weight), FR (Filling rate), ST (Shell thickness), FC (Fat content), PC (Protein content), respectively. Figure S2. The distribution of SNPs on 16 chromosomes mapped to reference genome. Figure S3. The distribution of InDels on 16 chromosomes mapped to reference genome. Figure S4. The population structure of 101 walnut accessions from K=2 to K=4 by using the software Admixture v1.3. Figure S5. Principal component analysis (PCA) of 101 walnut accessions. PCA analysis based on (A) PC1 (6.75%) and PC2 (3.65%), (B) PC2 (3.65%) and PC3 (2.85%), and (C) PC1 (6.75%) and PC3 (2.85%), respectively. Figure S6. Cluster analysis on phenotypic traits of 101 walnut accessions. Clustering based on methods of (A) Neighbor-joining (NJ) and (B) unweighted pair group method with arithmetic mean (UPGMA). Figure S7. The Manhattan plot of the 10 phenotypic association results and the corresponding Q-Q plot. The results are calculated by the GEMMA software, and the grey dotted line represents the significant threshold. (A). CD; (B). FC; (C). FI; (D). FR; (E). LD; (F). NW; (G). PC; (H). SD; (I). ST; (J). SW. Figure S8. (A) The GO analysis of associated genes in shell thickness. (B) The KEGG enrichment analysis of associated genes in shell thickness. Figure S9. The protein sequence alignment of the PXC1 gene homologs in 11 plants. [file 12870_2022_3824_MOESM1_ESM.docx]
